# Comparative transcriptome profiling analyses during the lag phase uncover *YAP1*, *PDR1, PDR3, RPN4*, and *HSF1 *as key regulatory genes in genomic adaptation to the lignocellulose derived inhibitor HMF for *Saccharomyces cerevisiae*

**DOI:** 10.1186/1471-2164-11-660

**Published:** 2010-11-24

**Authors:** Menggen Ma, Z Lewis Liu

**Affiliations:** 1 Bioenergy Research Unit, National Center for Agricultural Utilization Research, USDA-ARS, Peoria, IL USA

## Abstract

**Background:**

The yeast *Saccharomyces cerevisiae *is able to adapt and *in situ *detoxify lignocellulose derived inhibitors such as furfural and HMF. The length of lag phase for cell growth in response to the inhibitor challenge has been used to measure tolerance of strain performance. Mechanisms of yeast tolerance at the genome level remain unknown. Using systems biology approach, this study investigated comparative transcriptome profiling, metabolic profiling, cell growth response, and gene regulatory interactions of yeast strains and selective gene deletion mutations in response to HMF challenges during the lag phase of growth.

**Results:**

We identified 365 candidate genes and found at least 3 significant components involving some of these genes that enable yeast adaptation and tolerance to HMF in yeast. First, functional enzyme coding genes such as *ARI1, ADH6, ADH7*, and *OYE3*, as well as gene interactions involved in the biotransformation and inhibitor detoxification were the direct driving force to reduce HMF damages in cells. Expressions of these genes were regulated by *YAP1 *and its closely related regulons. Second, a large number of PDR genes, mainly regulated by *PDR1 *and *PDR3*, were induced during the lag phase and the PDR gene family-centered functions, including specific and multiple functions involving cellular transport such as *TPO1, TPO4, RSB1, PDR5, PDR15, YOR1*, and *SNQ2*, promoted cellular adaptation and survival in order to cope with the inhibitor stress. Third, expressed genes involving degradation of damaged proteins and protein modifications such as *SHP1 *and *SSA4*, regulated by *RPN4*, *HSF1*, and other co-regulators, were necessary for yeast cells to survive and adapt the HMF stress. A deletion mutation strain *Δrpn4 *was unable to recover the growth in the presence of HMF.

**Conclusions:**

Complex gene interactions and regulatory networks as well as co-regulations exist in yeast adaptation and tolerance to the lignocellulose derived inhibitor HMF. Both induced and repressed genes involving diversified functional categories are accountable for adaptation and energy rebalancing in yeast to survive and adapt the HMF stress during the lag phase of growth. Transcription factor genes *YAP1*, *PDR1, PDR3, RPN4*, and *HSF1 *appeared to play key regulatory rules for global adaptation in the yeast *S. cerevisiae*.

## Background

Bioethanol production from lignocellulosic biomass including agricultural and forestry residues has attracted increased attention worldwide [1-8]. Lignocellulosic biomass needs to be depolymerized into simple sugars in order to be utilized for microbial fermentation. The commonly applied dilute acid pretreatment generates numerous chemical byproducts that inhibit cell growth and interfere with subsequent microbial fermentation [5,9-11]. Among numerous inhibitory compounds, furfural and 5-hydroxymethylfurfural (HMF) are commonly encountered inhibitors [9,12-14]. Furfural and HMF are formed by dehydration of pentoses and hexoses released from hemicellulose and cellulose, respectively [15,16]. These inhibitors can damage cell structures, inhibit cell growth, reduce enzymatic activities, generate cellular reactive oxygen species (ROS), break down DNA, and inhibit protein and RNA synthesis [14,17-20]. The presence of fermentation inhibitors represents a bottle neck in cellulosic ethanol conversion technology and overcoming the inhibitor effect is one of the fundamental challenges to the industrial production of bioethanol from lignocellulosic biomass.

Furfural and its conversion product have been widely studied while knowledge of HMF conversion is limited due to a lack of commercial source of its conversion product [5,14,15,21-23]. Unlike evaporative furfural, HMF is more stable and difficult to degrade in cell culture. Recently, an HMF metabolic conversion product was isolated and identified as 2, 5-bis-hydroxymethylfuran (Furan-2,5-dimethanol, FDM) [24]. A dose-dependent response of yeast to HMF was demonstrated and a lag phase was used to measure levels of strain tolerance [24,25]. The yeast *Saccharomyces cerevisiae *is able to *in situ *detoxify HMF into the less toxic compound FDM through NADPH-dependent reductions [24,26,27]. Typically, yeast strains show a lag of delayed cell growth after inhibitor challenge such as with furfural and HMF, under sublethal doses. Once HMF and furfural inhibitor levels were chemically reduced to a certain lower concentration, cell growth recovered and the glucose-to-ethanol conversion accelerated at a faster rate than would normally occur [24]. It was suggested that genomic adaptation occurred during the lag phase [23,28]. In fact, inhibitor-tolerant yeast strains showed significant shorter lag phases under the inhibitor challenges compared with a wild type strain [28,29]. Gene expressions of selected pathways of the tolerant yeast are distinct from the wild type control [29]. Sequence mutations are common and a large number of single nucleotide polymorphism (SNP) mutations were observed throughout all 16 chromosomes for a tolerant yeast strain (Liu et al, unpublished data; Xu, personal communication 2010). Adaptations appear to occur at the genome level. However, little is known about gene expression response and regulatory events for yeast during the adaptation lag phase. The objective of this study was to characterize transcriptome response of yeast during the lag phase after the HMF challenge. Using a comparative time course study, we investigated the dynamics of transcriptome profiling during this critical stage applying DNA microarray assays and regulatory analysis. Important genes, together with transcription factors (TFs) involved in the HMF stress response, were identified. The functions of selective candidate genes were verified by corresponding gene deletion mutation strains. Significant regulatory interaction networks were uncovered during the genome adaptation in yeast. Results of this study provide insight into mechanisms of yeast adaptation and tolerance to lignocellulose derived inhibitors. This will directly aid engineering efforts for more tolerant strain development.

## Results

### Cell growth response and metabolic conversion profiles

**Figure 1:**
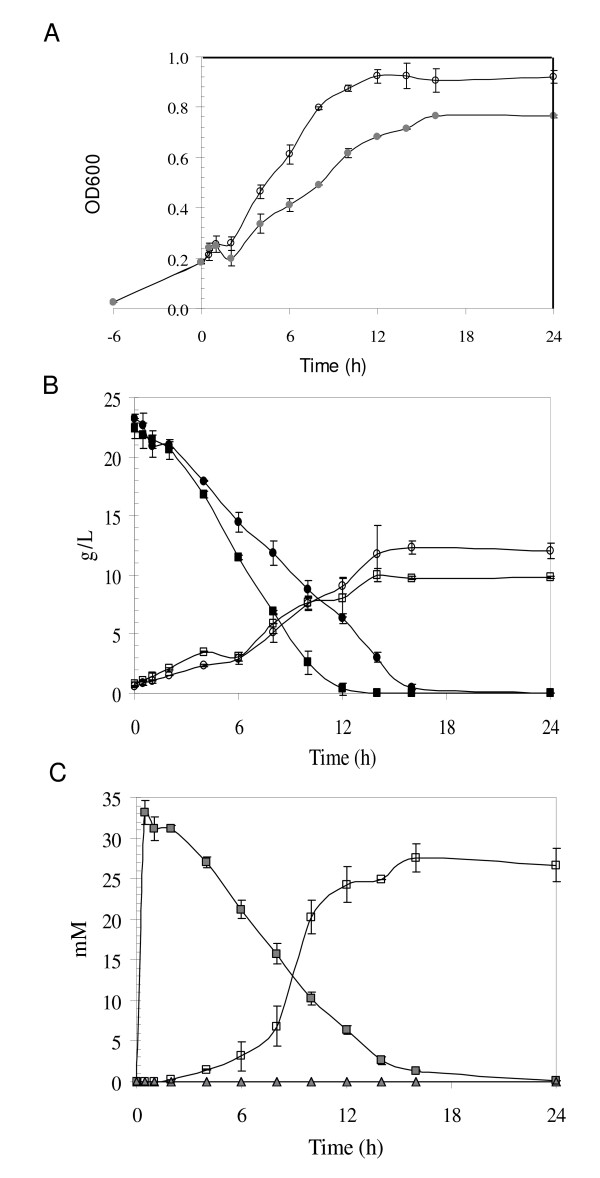
**Yeast growth and metabolic profile response to HMF**. Comparisons of cell growth and metabolic profiles of *Saccharomyces cerevisiae *NRRL Y-12632 between an HMF treatment (30 mM) and an untreated condition. A. Cell growth as measured by OD_600 _for HMF treated condition (grey circle) and control (open circle). B. Glucose consumption (filled circle) and ethanol conversion (open circle) for HMF treated condition versus glucose (filled square) and ethanol (open square) for control. C. HMF (grey square) and its conversion product furandimethanol (FDM) (open square) for HMF treated condition versus HMF (grey triangle) and FDM (open triangle) for the control.

Compared to a non-treated control, yeast challenged by HMF displayed a significant drop in cell growth as measured by OD_600 _absorbance 2 h after the treatment (Figure 1A). Although the cell growth was recovered at a later time, cell density of the HMF-treated yeast was relatively low throughout the course of the study. Similarly, glucose consumption for the HMF-treated culture was slower and glucose was depleted at 16 h, approximately 4 h later than the non-treated control (Figure 1B). As expected, HMF was undetectable and FDM was detected as HMF conversion product [24] in HMF-treated cultures less than 24 h after incubation (Figure 1C). No HMF or FDM was detected from the control culture.

### Transcription expression dynamics during the lag phase

**Figure 2:**
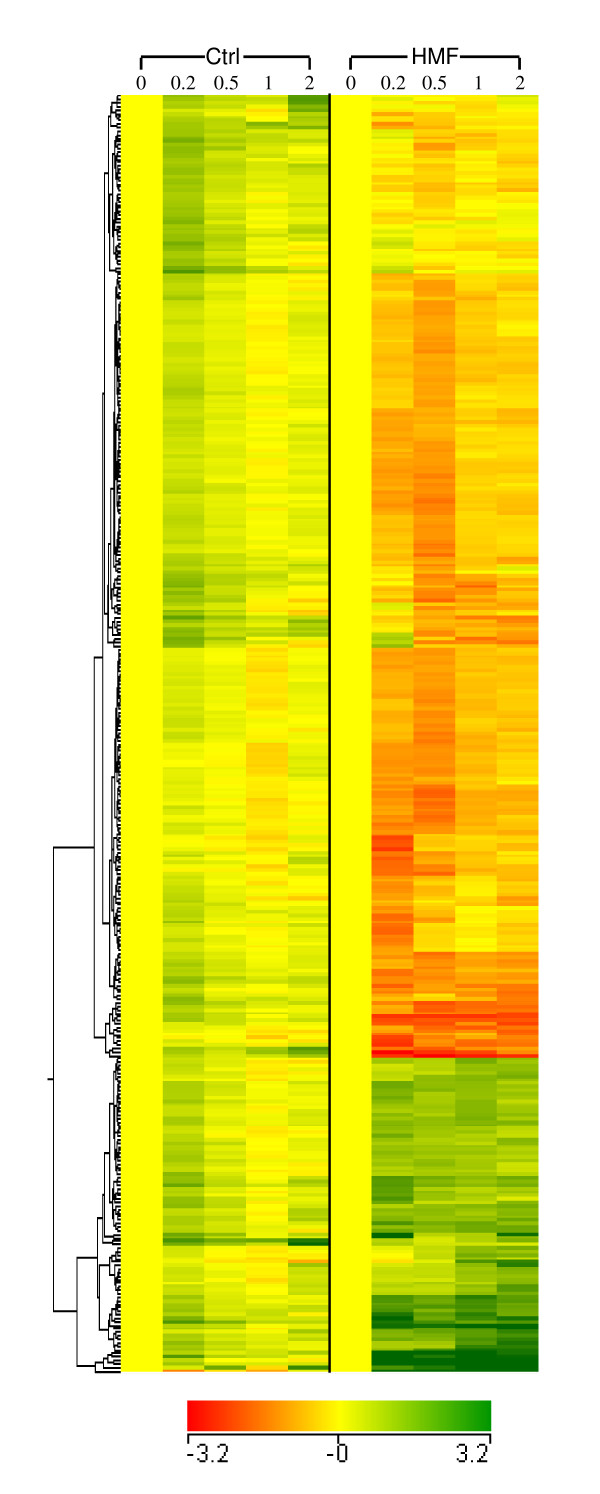
**Transcriptome response to HMF during the lag phase**. Hierarchical clustering of genes showing significant differential expression under HMF stress and displaying a 2-fold change for at least one time point compared with 0 h during the lag phase. Scales of the expression are indicated by an integrated color bar at the bottom.

Clustering analysis distinguished significant differences for expression responses by HMF between the treated and untreated conditions over time (data not shown). Among the more than 6,000 genes of the yeast genome, 365 genes were identified as differentially expressed by ANOVA for at least 2-fold changes during the lag phase of 10 to 120 min by the HMF challenge (Figure 2, Additional file 1). Among these, 71 genes were induced constantly throughout the lag phase while 246 genes were repressed at various stages of the lag phase (Figure 2, Table 1). Many of the induced genes showed immediate enhanced expressions within 10 min after the HMF challenge. These genes mainly fall with functional categories of reductase, pleiotropic drug resistance (PDR), proteasome and ubiquitin, amino acids metabolism, stress response functions, and others (Table 1 and 2). For example, *ADH7*, encoding NADPH-dependent medium chain alcohol dehydrogenase displayed the highest induction of more than 30-fold increase in mRNA abundance at 10 min after the HMF treatment. Other significantly induced genes including *ARI1*, *GRE2*, *PDR5*, *RSB1*, *PUT1*, *CHA1*, *HSP26*, *SSA4*, and *OYE3*, which showed more than 10-fold mRNA increase at various times during the lag phase.

**Table 1:** Significantly induced genes of Saccharomyces cerevisiae by HMF during the lag phase

Systematic Name	Standard Name	Description	Fold change			
			
			0.2 h	0.5 h	1 h	2 h
Reductase						
YCR105W	*ADH7*	NADPH-dependent medium chain alcohol dehydrogenase with broad substrate specificity	+38.4	+60.3	+81.3	+39.5
YGL157W	*ARI1*	NADPH-dependent aldehyde reductase	+12.3	+21.3	+29.2	+27.2
YOL151W	*GRE2*	3-methylbutanal reductase and NADPH-dependent methylglyoxal reductase (D-lactaldehyde dehydrogenase)	+8.0	+7.6	+10.4	+12.0
YOR374W	*ALD4*	NAD(P)-dependent mitochondrial aldehyde dehydrogenase	+4.3	+3.2	+2.7	+3.2
PDR family						
YOR153W	*PDR5*	Plasma membrane ATP-binding cassette (ABC) transporter	+30.0	+19.0	+30.6	+23.7
YPL058C	*PDR12*	Plasma membrane ATP-binding cassette (ABC) transporter	+7.1	+4.1	+6.2	+3.3
YDR406W	*PDR15*	Plasma membrane ATP binding cassette (ABC) transporter	+9.5	+3.5	+5.4	+7.3
YDR011W	*SNQ2*	Plasma membrane ATP-binding cassette (ABC) transporter	+4.7	+3.8	+6.4	+4.1
YGR281W	*YOR1*	Plasma membrane ATP-binding cassette (ABC) transporter	+4.7	+3.0	+4.4	+3.5
YOR049C	*RSB1*	Suppressor of sphingoid long chain base (LCB) sensitivity of an LCB-lyase mutation	+15.7	+6.9	+9.5	+8.6
YLR099C	*ICT1*	Lysophosphatidic acid acyltransferase	+3.4	+2.9	+4.8	+6.2
YER142C	*MAG1*	3-methyl-adenine DNA glycosylase involved in protecting DNA against alkylating agents	+4.3	+4.5	+5.1	+3.7
YER143W	*DDI1*	DNA damage-inducible v-SNARE binding protein	+2.4	+2.2	+2.4	+1.7
YLL028W	*TPO1*	Polyamine transporter that recognizes spermine, putrescine, and spermidine	+4.5	+3.3	+5.1	+4.3
YOR273C	*TPO4*	Polyamine transporter that recognizes spermine, putrescine, and spermidine	+2.7	+2.0	+3.0	+2.6
YDL020C	*RPN4*	Transcription factor that stimulates expression of proteasome genes	+1.9	+2.5	+2.8	+3.3
YGR035C	YGR035C	Putative protein of unknown function	+3.1	+3.8	+4.1	+5.9
YLL056C	YLL056C	Putative protein of unknown function	+2.0	+2.0	+5.8	+6.2
YMR102C	YMR102C	Putative protein of unknown function	+1.8	+1.6	+2.2	+2.9
Proteasome and ubiquitin						
YER012W	*PRE1*	Beta 4 subunit of the 20 S proteasome	+2.7	+2.4	+2.1	+1.8
YJL001W	*PRE3*	Beta 1 subunit of the 20 S proteasome	+3.0	+3.0	+2.6	+2.1
YOL038W	*PRE6*	Alpha 4 subunit of the 20 S proteasome	+2.0	+2.2	+2.8	+2.3
YBL041W	*PRE7*	Beta 6 subunit of the 20 S proteasome	+3.5	+2.6	+2.6	+1.9
YOR362C	*PRE10*	Alpha 7 subunit of the 20 S proteasome	+2.4	+2.2	+2.2	+1.9
YER094C	*PUP3*	Beta 3 subunit of the 20 S proteasome involved in ubiquitin-dependent catabolism	+2.4	+2.2	+2.9	+2.1
YDR427W	*RPN9*	Non-ATPase regulatory subunit of the 26 S proteasome	+2.7	+2.4	+2.4	+2.1
YFR052W	*RPN12*	Subunit of the 19 S regulatory particle of the 26 S proteasome lid	+2.8	+2.4	+2.6	+2.5
YHL030W	*ECM29*	Major component of the proteasome	+4.6	+3.3	+3.6	+2.6
YDL007W	*RPT2*	One of six ATPases of the 19 S regulatory particle of the 26 S proteasome involved in the degradation of ubiquitinated substrates	+3.3	+2.6	+2.6	+2.4
YDR394W	*RPT3*	One of six ATPases of the 19 S regulatory particle of the 26 S proteasome involved in the degradation of ubiquitinated substrates	+2.9	+2.0	+2.5	+2.5
YOR259C	*RPT4*	One of six ATPases of the 19 S regulatory particle of the 26 S proteasome involved in the degradation of ubiquitinated substrates	+3.1	+2.6	+3.2	+2.5
YBL058W	*SHP1*	UBX (ubiquitin regulatory X) domain-containing protein that regulates Glc7p phosphatase activity and interacts with Cdc48p	+2.5	+2.0	+2.1	+2.8
YFL044C	*OTU1*	Deubiquitylation enzyme that binds to the chaperone-ATPase Cdc48p	+2.3	+1.9	+3.3	+2.3
Amino acids						
YLR142W	*PUT1*	Proline oxidase	+4.8	+6.9	+10.8	+43.0
YHR037W	*PUT2*	Delta-1-pyrroline-5-carboxylate dehydrogenase	+3.7	+3.7	+4.7	+3.3
YJR010W	*MET3*	ATP sulfurylase	+2.5	+2.7	+3.3	+3.6
YKL001C	*MET14*	Adenylylsulfate kinase	+1.7	+2.6	+2.8	+3.2
YCL064C	*CHA1*	Catabolic L-serine (L-threonine) deaminase, catalyzes the degradation of both L-serine and L-threonine	+13.3	+17.7	+15.8	+7.3
YLR089C	*ALT1*	Alanine transaminase	+2.9	+3.1	+4.1	+4.9
YPL111W	*CAR1*	Arginase, responsible for arginine degradation	+3.4	+2.2	+1.9	+1.6
Stress						
YBR072W	*HSP26*	Small heat shock protein (sHSP) with chaperone activity	+10.8	+1.8	+2.0	+8.0
YER103W	*SSA4*	Heat shock protein that is highly induced upon stress	+13.2	+1.5	+2.0	+2.6
Transcription factors						
YML007W	*YAP1*	Basic leucine zipper (bZIP) transcription factor required for oxidative stress tolerance	+3.3	+2.1	+2.5	+2.2
YDL020C	*RPN4*	Transcription factor that stimulates expression of proteasome genes	+1.9	+2.5	+2.8	+3.3
Others						
YPL171C	*OYE3*	Conserved NADPH oxidoreductase containing flavin mononucleotide (FMN)	+5.9	+3.9	+5.3	+17.7
YOR306C	*MCH5*	Plasma membrane riboflavin transporter	+2.6	+4.9	+7.8	+7.2
YAR073W	*IMD1*	Nonfunctional protein with homology to IMP dehydrogenase	+2.2	+2.1	+3.4	+2.7
YBL078C	*ATG8*	Component of autophagosomes and Cvt vesicles	+4.5	+3.3	+2.7	+3.5
YBR062C	YBR062C	Hypothetical protein	+2.5	+2.4	+2.4	+2.1
YML130C	*ERO1*	Thiol oxidase required for oxidative protein folding in the endoplasmic reticulum	+2.3	+2.4	+2.8	+2.3
YBR114W	*RAD16*	Protein that recognizes and binds damaged DNA in an ATP-dependent manner (with Rad7p) during nucleotide excision repair	+2.8	+2.1	+2.8	+1.7
YBR170C	*NPL4*	Endoplasmic reticulum and nuclear membrane protein	+2.4	+2.7	+2.2	+1.5
YDL021W	*GPM2*	Homolog of Gpm1p phosphoglycerate mutase	+3.5	+1.6	+1.7	+2.8
YBL101W-A	YBL101W-A	Retrotransposon TYA Gag gene co-transcribed with TYB Pol	+1.4	+1.6	+2.6	+6.4
YDR210W-B	YDR210W-B	Retrotransposon TYA Gag and TYB Pol genes	+1.9	+1.7	+2.6	+4.4
YDR316W-B	YDR316W-B	Retrotransposon TYA Gag and TYB Pol genes	+1.8	+1.8	+2.6	+3.2
YDR365W-B	YDR365W-B	Retrotransposon TYA Gag and TYB Pol genes	+1.7	+1.7	+2.2	+4.3
YDR515W	*SLF1*	RNA binding protein that associates with polysomes	+2.7	+2.7	+2.4	+2.7
YOR009W	*TIR4*	Cell wall mannoprotein of the Srp1p/Tip1p family of serine-alanine-rich proteins	-1.3	+1.3	+4.6	+5.9
YPL156C	*PRM4*	Pheromone-regulated protein proposed to be involved in mating	+3.4	+2.8	+3.5	+3.5
YGL062W	*PYC1*	Pyruvate carboxylase isoform	+2.3	+2.6	+3.1	+2.1
YOR007C	*SGT2*	Glutamine-rich cytoplasmic protein of unknown function	+2.8	+2.5	+2.9	+3.0
YOR052C	YOR052C	Nuclear protein of unknown function	+4.1	+2.6	+1.8	+2.4
YDR034W-B	YDR034W-B	Protein of unknown function	+7.2	+5.0	+3.6	+4.5
YML125C	*PGA3*	Putative cytochrome b5 reductase	+1.6	+2.7	+3.0	+2.4
YBL107C	YBL107C	Putative protein of unknown function	+2.4	+2.6	+2.1	+2.9
YBR255C-A	YBR255C-A	Putative protein of unknown function	+4.0	+2.0	+1.8	+2.1
YER137C	YER137C	Putative protein of unknown function	+2.9	+2.4	+2.5	+3.6
YGR111W	YGR111W	Putative protein of unknown function	+2.1	+2.2	+4.3	+7.1
YHR138C	YHR138C	Putative protein of unknown function	+4.2	+2.8	+2.4	+2.8
YKR011C	YKR011C	Putative protein of unknown function	+4.6	+2.3	+1.9	+1.3
YNL155W	YNL155W	Putative protein of unknown function	+3.7	+2.4	+2.2	+1.9
YOR059C	YOR059C	ORF, Uncharacterized	+2.1	+2.6	+2.2	+2.2

**Table 2:** Gene Ontology (GO) categories and terms for significantly induced genes by HMF during the lag phase in Saccharomyces cerevisiae

GO ID	GO term	Gene(s)
Cellular component		
GO:0005737	Cytoplasm	SHP1*, ***ATG8***, YBL107C, ***HSP26***, ***NPL4***, ***CHA1***, GPM2, ***SNQ2***, ***RPN9***, ***SLF1***, ***SSA4***, ***OTU1***, ***RPN12***, ***PYC1***, ***ARI1***, ***YGR111W***, ***ECM29***, ***PUT2***, ***PRE3***, ***MET3***, ***MET14***, ***TPO1***, ***ALT1***, ***PUT1***, ***YAP1***, ***PGA3***, ***ERO1***, ***YNL155W***, ***PRE6***, ***GRE2***, SGT2, ***RSB1***, YOR059C, ***PDR5***, ***TPO4***, ***PRE10***, ***ALD4***, ***CAR1***
GO:0005634	Nucleus	***SHP1***, ***YBL100W-A***, ***HSP26***, ***RAD16***, ***RPT2***, ***RPN4***, ***YDR210W-B***, ***YDR316W-B***, ***YDR365W-B***, ***PRE1***, ***SSA4***, ***MAG1***, ***OTU1***, ***ARI1***, ***YGR111W***, ***ECM29***, YKR011C, ***YAP1***, ***YNL155W***, ***GRE2***, YOR052C, ***RPT4***
GO:0016020	Membrane	***ATG8***, ***NPL4***, ***SNQ2***, ***PDR15***, ***DDI1***, ***YOR1***, ***TPO1***, ***PGA3***, ***RSB1***, ***PDR5***, ***TPO4***, ***MCH5***, ***PDR12***, *PRM4*
GO:0005575	Cellular component unknown	*IMD1, YBR062C, YBR255C-A, YDR034W-B, YER137C, YGR035C, **YHR138C**, YLL056C, ICT1, OYE3*
GO:0005886	Plasma membrane	***SNQ2***, ***DDI1***, ***YOR1***, ***TPO1***, ***PGA3***, ***RSB1***, ***PDR5***, ***TPO4***, ***MCH5***, ***PDR12***
GO:0005739	Mitochondrion	***CHA1***, ***SNQ2***, ***PUT2***, ***MET3***, ***ALT1***, ***PUT1***, ***PRE6***, ***PDR5***, ***ALD4***
GO:0005783	Endoplasmic reticulum	***NPL4***, ***PGA3***, ***ERO1***, ***RSB1***
GO:0005773	Vacuole	***ATG8***, ***TPO1***, ***TPO4***
GO:0005624	Membrane fraction	***SNQ2***, ***YOR1***
GO:0005933	Cellular bud	***TPO1***
GO:0005618	Cell wall	*TIR4*
GO:0012505	Endomembrane system	***NPL4***
GO:0030427	Cite of polarized growth	***CAR1***
Other	Other	***PRE7***, *ADH7*, ***RPT3***, ***PUP3***
Biological process		
GO:0008150	Biological process unknown	*IMD1, YBL107C, YBR062C, YBR255C-A, GPM2, YDR034W-B, YER137C, **ARI1**, YGR035C, YKR011C, YLL056C, **YNL155W**, TIR4, YOR052C, YOR059C, PRM4, OYE3*
GO:0044257	Cellular protein catabolic process	***PRE7***, ***SHP1***, ***RAD16***, ***NPL4***, ***RPT2***, ***RPT3***, ***RPN9***, ***PRE1***, ***PUP3***, ***DDI1***, ***RPN12***, ***PRE3***, ***PRE6***, ***RPT4***, ***PRE10***
GO:0006810	Transport	***ATG8***, ***PDR15***, ***SSA4***, ***DDI1***, ***YOR1***, ***TPO1***, ***PGA3***, ***RSB1***, ***PDR5***, ***TPO4***, ***MCH5***, ***PDR12***
GO:0006950	Response to stress	***ATG8***, ***HSP26***, ***RAD16***, ***RPN4***, ***SNQ2***, ***PRE1***, ***SSA4***, ***MAG1***, ***PRE3***, ***YAP1***, *SGT2*
GO:0042221	Response to chemical stimulus	***RPN4***, ***SNQ2***, ***PDR15***, ***YOR1***, ***MET14***, ***YAP1***, ***PDR5***
GO:0006519	Cellular amino acid and derivative metabolic process	***CHA1***, ***PUT2***, ***MET3***, ***MET14***, ***ALT1***, ***PUT1***, ***CAR1***
GO:0032196	Transposition	***YBL100W-A***, ***YDR210W-B***, ***YDR316W-B***, ***YDR365W-B***
GO:0006457	Protein folding	***HSP26***, ***SSA4***, ***ERO1***
GO:0006350	Transcription	***RPN4***, ***OTU1***, ***YAP1***
GO:0006464	Protein modification process	***RAD16***, ***OTU1***, ***ERO1***
GO:0030435	Sporulation resulting in formation of a cellular spore	***SHP1***, ***PRE1***, ***PRE3***
GO:0006259	DNA metabolic process	***RAD16***, ***RPN4***, ***MAG1***
GO:0016044	Membrane organization	***ATG8***, ***YHR138C***, ***RSB1***
GO:0007033	Vacuole organization	***ATG8***, ***YHR138C***
GO:0044262	Cellular carbohydrate metabolic process	***SHP1***, ***PYC1***
GO:0044255	Cellular lipid metabolic process	*ICT1, **GRE2***
GO:0006766	Vitamin metabolic process	***PYC1***, ***ALD4***
GO:0046483	Heterocycle metabolic process	***PUT2***, ***PUT1***
GO:0051186	Cofactor metabolic process	***PYC1***, ***ALD4***
GO:0016192	Vesicle-mediated transport	***ATG8***, ***DDI1***
GO:0051276	Chromosome organization	***RAD16***
GO:0016070	RNA metabolic process	***YAP1***
GO:0006412	Translation	***SLF1***
GO:0006091	Generation of precursor metabolites and energy	***SHP1***
GO:0070271	Protein complex biogenesis	***RPN9***
GO:0007049	Cell cycle	***RPN4***
GO:0019725	Cellular homeostasis	***SLF1***
Other	Other	*ADH7, **YGR111W**, **ECM29***
Molecular function		
GO:0016787	Hydrolase activity	***PRE7***, ***RAD16***, ***RPT2***, ***SNQ2***, ***YDR210W-B***, ***YDR316W-B***, ***YDR365W-B***, ***RPT3***, ***PDR15***, ***PRE1***, ***PUP3***, ***SSA4***, ***MAG1***, ***OTU1***, ***RPN12***, ***YOR1***, ***PRE3***, ***PRE6***, ***RSB1***, ***PDR5***, ***RPT4***, ***PRE10***, ***PDR12***, ***CAR1***
GO:0003674	Molecular function unknown	*IMD1, **ATG8**, YBL107C, YBR062C, **NPL4**, YBR255C-A, GPM2, YDR034W-B, YER137C, YGR035C, **YGR111W**, YKR011C, YLL056C, **PGA3**, **YNL155W**, SGT2, TIR4, YOR052C, YOR059C, PRM4*
GO:0008233	Peptidase activity	***PRE7***, ***RPT2***, ***YDR210W-B***, ***YDR316W-B***, ***YDR365W-B***, ***RPT3***, ***PRE1***, ***PUP3***, ***OTU1***, ***RPN12***, ***PRE3***, ***PRE6***, ***RPT4***, ***PRE10***
GO:0005215	Transporter activity	***SNQ2***, ***PDR15***, ***YOR1***, ***TPO1***, ***RSB1***, ***PDR5***, ***TPO4***, ***MCH5***, ***PDR12***
GO:0016491	Oxidoreductase activity	*ADH7, **ARI1**, **PUT2**, **PUT1**, **ERO1**, **GRE2**, **ALD4**, OYE3*
GO:0005515	Protein binding	***YBL100W-A***, ***HSP26***, ***YDR210W-B***, ***YDR316W-B***, ***YDR365W-B***, ***SSA4***, ***DDI1***, ***ECM29***
GO:0016740	Transferase activity	***YDR210W-B***, ***YDR316W-B***, ***YDR365W-B***, ***MET3***, ***MET14***, ***ALT1***, *ICT1*
GO:0003723	RNA binding	***YBL100W-A***, ***YDR210W-B***, ***YDR316W-B***, ***YDR365W-B***, ***SLF1***
GO:0016779	Nucleotidyltransferase activity	***YDR210W-B***, ***YDR316W-B***, ***YDR365W-B***, ***MET3***
GO:0003677	DNA binding	***RAD16***, ***RPN4***, ***YAP1***
GO:0016874	Ligase activity	***RAD16***, ***PYC1***
GO:0030528	Transcription regulator activity	***RPN4***, ***YAP1***
GO:0030234	Enzyme regulator activity	***SHP1***, ***YHR138C***
GO:0016829	Lyase activity	***CHA1***
GO:0005198	Structural molecule activity	***RPN9***
GO:0016853	Isomerase activity	*GPM2*

* Genes in bold indicate their encoding proteins or enzymes are involved in more than one function

**Figure 3:**
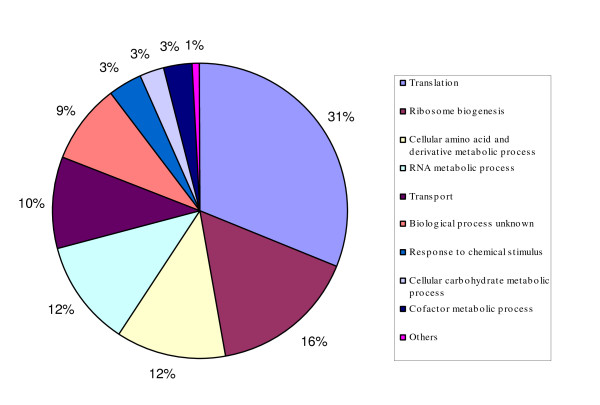
**Functional categories of repressed gene expression**. Distribution of functional categories of repressed gene expressions by HMF treatment.

The repressed genes are mainly involved in the functional categories of ribosome biogenesis, amino acid and derivative metabolic process, RNA metabolic process, transport, and others (Figure 3, Additional file 2). Most of the genes encoding enzymes for arginine biosynthesis were severely repressed, such as *ARG1*, *ARG3*, *ARG4*, *ARG5,6*, *ARG7*, and *ARG8 *(Additional file 1). For the repressed genes, three types of dynamic responses were observed. A small group of two dozen genes showed transient inductions at 10 min but quickly turned into repressed after 30 min, such as *PCL6 *and *PCL8 *for glycogen metabolism, *MAL1*, *MAL11*, and *MPH3 *for maltose utilization. Another group of about 30 genes were constantly repressed, and these were mainly in the functional categories of amino acid metabolism, such as *ARG1*, *ARG3*, *ARG4*, *ARG5,6*, *ARG7 *for arginine metabolism, *HIS1*, *HIS3*, and *HIS4 *for histidine metabolism, *ARO3*, *ARO4*, *HOM2*, and *HOM3 *for aromatic amino acid metabolism. The third group of the repressed genes were initially repressed at 10 or 30 min but recovered at later time points. This group of repressed genes fall within the categories of rRNA processing, tRNA export, and ribosomal biogenesis such as *NOB1*, *PUS1*, *RRP5*, *NOP56*, and *CBF5*; mitochondrial mRNA maturase such as *BI2 *and *BI3*; vitamin B6 biosynthesis gene *SNZ1*; and telomere length maintenance gene *YKU80 *(Additional file 1).

### Relevant transcription factors

**Figure 4:**
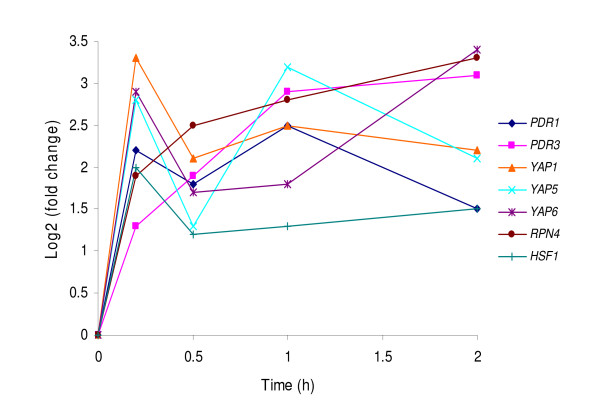
**Expression response of important transcription factor genes**. Expression patterns of seven selective genes encoding important transcription factors for positively regulating gene expression response to HMF stress.

Under the HMF challenge, we found that seven transcription factor genes, *PDR1*, *PDR3*, *YAP1*, *YAP5*, *YAP6*, *RPN4*, and *HSF1*, displayed significant greater expression during the lag phase in response to the HMF challenge (Figure 4). Except for *HSF1*, most transcription factor genes displayed greater than 2-fold increase after the HMF treatment. By the aid of T-profiler [30], YEASTRACT database [31] and interactive pathway analysis using GeneSpring GX 10.0, we identified these genes as the most important transcription factor genes positively regulating gene expression response in adaptation to the HMF stress during the lag phase in yeast.

**Figure 5:**
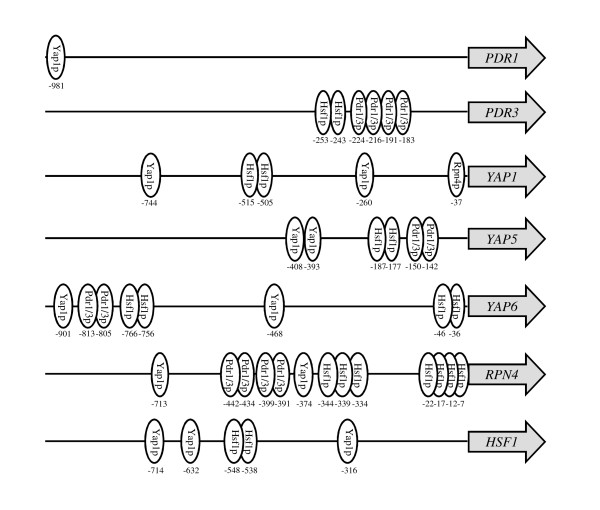
**DNA binding sites in promoter region**. DNA binding sites for seven selective transcription factor genes *YAP1*, *YAP5*, *YAP6*, *PDR1*, *PDR3*, *RPN4*, and *HSF1 *in the promoter regions (from -1000 to -1)analyzed based on YEASTRACT.

We further analyzed protein binding motifs for these genes and found each transcription factor gene harbored protein binding motifs for Pdr1p, Pdr3p, Yap1p, Yap5p, Yap6p, Rpn4p, and Hsf1p. DNA binding motifs of Pdr1/3p were found in promoter regions of *PDR3*, *YAP5*, *PDR6*, and *RPN4*; Yap1p binding sites in all six transcription factor genes except for *PDR1*; and Hsf1p sites in all six genes except for *PDR1 *(Figure 5). Except for *PDR1 *which had a single Yap1p binding site, each of the other six transcription factor genes displayed multiple binding sites for multiple transcription factors. For example, *RPN4 *had 13 binding sites of 4 transcription factors, and *PDR3 *had 6 sites for 2. Interactions involving multiple transcription factors apparently exist. For example, highly expressed *RPN4 *in this study was found to be regulated by Yap1p, Pdr1p, Pdr3p, and Hsf1p that supported by ChIP-chip data and microarray assay of transcription factor mutations [32-37]. On the other hand, it also demonstrated positive feedback to its regulators of Yap1p and Pdr1p [33,37,38]. The presence of DNA binding motifs of a transcription factors' own in its promoter region, such as *PDR3*, *YAP1*, and *HSF1 *(Figure 5), suggested a self-regulated expression. The highly induced expression of the seven transcription factor genes in response to the HMF challenge and multiple protein binding motifs across the transcription factors suggested co-regulation and interactions of multiple transcription factors under the stress. As for many repressed expression responses to HMF, we identified five transcription factor genes *ARG80*, *ARG81*, *GCN4*, *FHL1*, and *RAP1 *that displayed down-regulated expressions (Additional file 3).

### YAP1 regulated gene expression networks

Among the seven transcription factor genes, *YAP1 *displayed consistently higher inductions, a 2- to 3-fold increase during the lag phase (Figure 4). Yap1p acts as a sensor for oxidative molecules, and activates the transcription response of anti-oxidant genes by recognizing Yap1p response elements (YRE), 5'-TKACTMA-3', in the promoter region [33,39,40].

**Figure 6:**
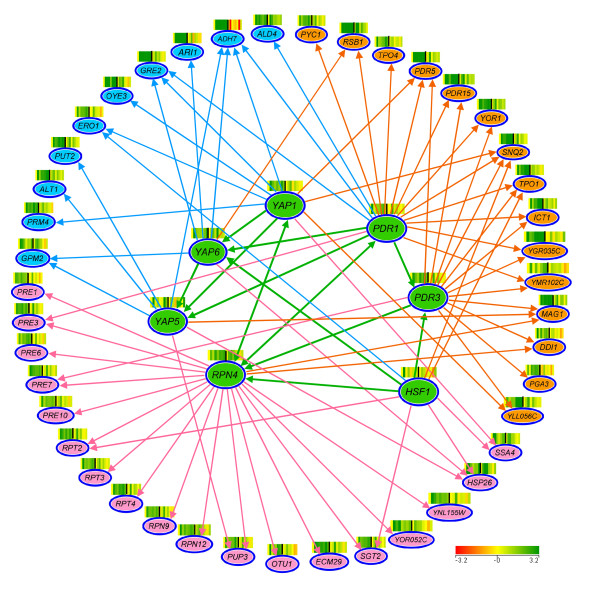
**Significant positive gene regulatory networks**. Regulatory interaction networks between transcription factors (filled green and green arrows) and induced genes for functional reduction enzymes (filled blue and blue arrows), PDR gene family (filled orange and orange arrows), and proteasome function (filled pink and pink arrows). Scales of the expression are indicated by an integrated color bar at the right bottom corner.

A total of 41 HMF-induced genes were found to have the YRE sequence in their promoter region (Additional file 4). Many genes were confirmed to be regulated directly by *YAP1 *or indirectly through *YAP5 *and *YAP6 *(Figure 6). Most *YAP1*-regulated genes were classified in the functional categories of redox metabolism, amino acid metabolism, stress response, DNA repair, and others (Table 2). For example, the highly induced oxidoreductase genes *ADH7*, *GRE2*, and *OYE3 *were found as regulons of *YAP1 *(Figure 6) [32,38,41]. *ADH7 *and *GRE2 *were also co-regulated by Yap5p and Yap6p [33,36]. These two genes were among those confirmed as reductases actively involved in the HMF detoxification [26]. *ARI1*, a recently characterized aldehyde reductase contributing to detoxification of furfural and HMF [27], was found to be regulated by Yap6p [33] which is a regulon of *YAP1*. In addition, *YAP1 *and other YAP gene family members were shown to co-regulate numerous genes in a wide range of functional categories such as PDR, heat shock protein, chaperones, amino acid metabolism, as well as other regulators.

**Figure 7:**
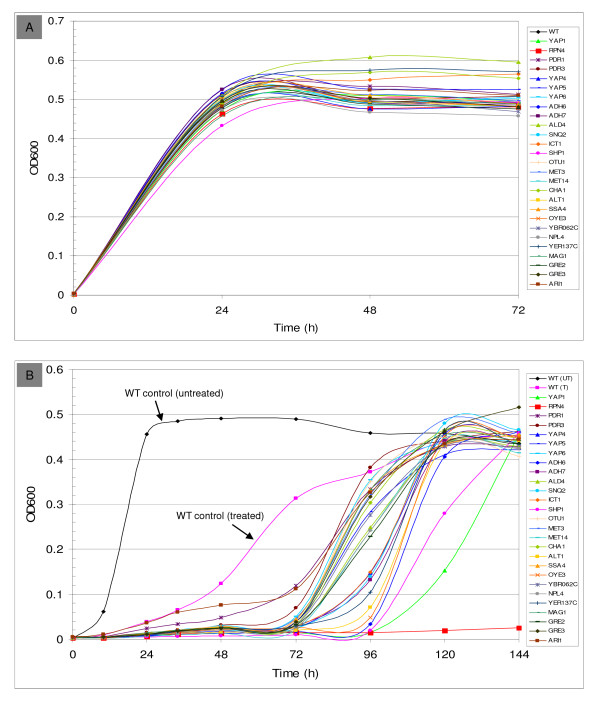
**Deletion mutants growth response to HMF**. Cell growth of deletion mutations and the parental wild type BY4742 (WT) on SC medium without HMF (A) and in the presence of 15 mM HMF (B) as measured by OD_600 _over time. Legend for each mutation is provided by a color code.

The significance of the role of the YAP gene family in adaptation and tolerance to HMF is confirmed by growth responses of the deletion mutations. Single YAP gene deletion mutations were able to grow normally without HMF treatment (Figure 7A). However, in the presence of 15 mM HMF, mutations *Δyap1, Δyap4, Δyap5*, and *Δyap6 *showed delayed growth compared with their parental strain (Figure 7B). Among these, *Δyap1 *displayed a 4-day long lag phase, indicating a profound functional defect affected by the *YAP1 *gene.

### PDR family and PDR1/3 involved regulatory interactions

Among the significantly induced genes by HMF, at least 15 genes were categorized into the PDR family (Table 1). Many genes displayed consistent induced expressions ranging from 3- to 30-fold increases during the lag phase (Table 1). Gene products of these increased transcripts were in the protein categories of drug/toxin transport for *TPO1 *and *TPO4*, Transport ATPase for *RSB1*, and ABC transporters for *PDR15 *(Table 3). *SNQ2*, *YOR1*, *PDR5*, and *PDR12 *encoding proteins shared functions of all these three categories. In addition, many PDR proteins have functions such as ATP binding and chemical agent resistance (Table 3). Most of these genes have the pleiotropic drug response element (PDRE) in their promoter regions (Additional file 4).

**Table 3:** Protein functional categories for significantly induced genes by HMF during the lag phase in Saccharomyces cerevisiae

MIPS ID	Functionary category	p-value	Entries
01 Metabolism			
01.01.03.03.02	Degradation of proline	7.82E-04	PUT2, PUT1*
01.01.03.05.02	Degradation of arginine	3.94E-04	**PUT1**, CAR1
01.02.03.01	Sulfate assimilation	3.54E-03	MET3, MET14
14 Protein fate (folding, modification, destination)			
14.07.11	Protein processing (proteolytic)	4.05E-09	**PRE7**, **ATG8**, **RPT2**, **RPT3**, **PRE1**, **PUP3**, **RPN12**, **PRE3**, **PRE6**, **RPT4**, **PRE10**
14.13	Protein/peptide degradation	3.97E-11	**PRE7**, SHP1, **ATG8**, NPL4, **RPT2**, RPN4, **RPT3**, RPN9, **PRE1**, **PUP3**, DDI1, OTU1, **RPN12**, ECM29, YHR138c, **PRE3**, **PRE6**, **RPT4**, **PRE10**
16 Protein with binding function or cofactor requirement (structural or catalytic)			
16.19.03	ATP binding	1.52E-03	**RPT2**, **SNQ2**, **RPT3**, **PDR15**, **YOR1**, **PDR5**, **RPT4**, **PDR12**
20 Cellular transport, transport facilities and transport routes			
20.01.27	Drug/toxin transport	4.70E-06	**SNQ2**, **YOR1**, TPO1, **PDR5**, TPO4, **PDR12**
20.03.22	Transport ATPases	3.68E-04	**SNQ2**, **YOR1**, RSB1, **PDR5**, **PDR12**
20.03.25	ABC transporters	1.44E-05	**SNQ2**, **PDR15**, **YOR1**, **PDR5**, **PDR12**
32 Cell rescue, defense and virulence			
32.05.01.03	Chemical agent resistance	1.73E-05	**SNQ2**, MAG1, **YOR1**, YAP1, **PDR5**

* Proteins in bold indicate functions involved in more than one category

HMF-induced transcription factor genes *PDR1 *and *PDR3 *regulate gene expression under a large variety of unrelated chemical stress conditions by binding to the PDRE of target genes [42-45]. Both Pdr1p and Pdr3p recognize CGG triplets oriented in opposite directions (CCGCGG) to form an inverted repeat [46], and able to form homodimers or heterodimers to activate target gene expression [42]. Many induced genes regulated by Pdr1p and/or Pdr3p in this group are involved in export of both xenobiotic compounds and endogenous toxic metabolites using ATP-binding cassette (ABC) transporters (Pdr5p, Pdr15p, Snq2p, and Yor1p), lipid composition of the plasma membrane (Rsb1p and Ict1p), export of polyamines by polyamine transporters (Tpo1p and Tpo4p), DNA repairing (Mag1p and Ddi1p), and other functions (Figure 6) [37,47-52]. At least eight genes induced by HMF in this study were regulated by both Pdr1p and Pdr3p. Pdr1p and Pdr3p also recognize and activate other subsets of genes. Pdr3p participates in certain processes that do not involve Pdr1p, such as regulating DNA damage-inducible genes *MAG1 *and *DDI1 *[53]. Similarly, some genes are only regulated by Pdr1p, such as *RSB1 *[54], *ADH7*, and *PRE3 *[32,33]. We also found that the *PDR3 *promoter contains two PDREs that can be autoregulated by itself in addition to being a regulon of Pdr1p [50,55]. *PDR1 *and *PDR3 *also demonstrated regulatory connections with a broad range of functional category genes as well as most active regulatory genes.

**Figure 8:**
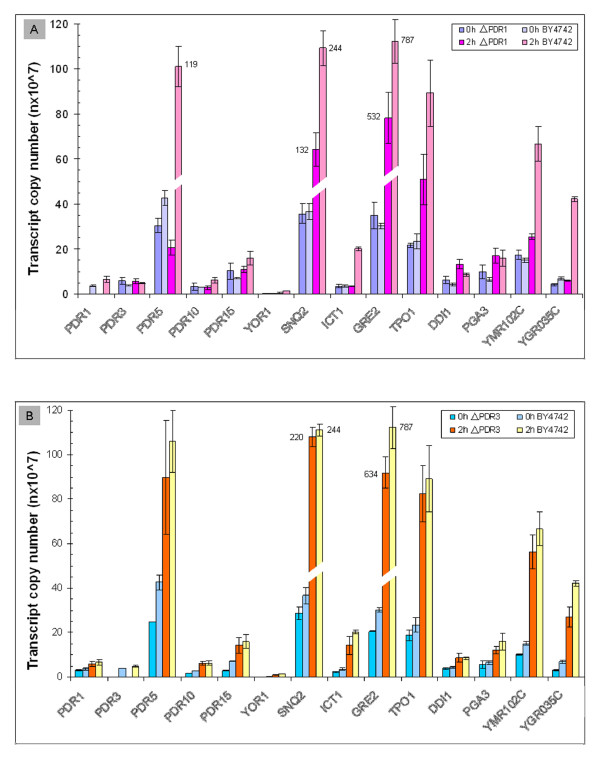
**The qRT-PCR for PDR gene family**. Expression abundance and gene interactions affected by deletion mutation *Δpdr1 *(A) and *Δpdr3 *(B) for selected PDR genes in response to HMF challenge compared with their parental wild type strain BY4742. Mean values are presented with error bars of standard deviations. Legend of value specificity is provided.

*PDR 1 *and *PDR3 *gene deletion mutations were assayed to confirm their influence on the expression of the potential regulons. When examined by qRT-PCR, mutant *Δpdr1 *displayed reduced transcriptional abundance for many genes, such as *PDR5, PDR10, PDR15, YOR1, SNQ2, ICT1, GRE2, TPO1, YMR102C*, and *YGR035C *compared with its parental strain BY4742 2 h after exposure to furfural and HMF (Figure 8A). The mutation *Δpdr3 *appeared to have a similar regulatory effect but to a lesser degree and to fewer genes (Figure 8B). However, it was clear that expression of *PGA3 *was affected by *Δpdr3 *but not *Δpdr1*.

### Regulatory interactions of RPN4 and HSF1

Among the genes induced by HMF, at least 14 ubiquitin-related and proteasome genes for protein degradation were identified (Figure 6). These genes, by encoding enzymes involving in the degradation of damaged proteins, maintain cell viability and functions under the inhibitor stress. The induction of these genes was predicted to be under the control of the transcription factor Rpn4p by binding to the proteasome-associated control element (PACE, 5'- GGTGGCAAA-3') [56], and the PACE was found in the promoter of most ubiquitin-related and proteasome genes induced by HMF (Additional file 4). In this study, *RPN4 *was continuously enhanced over time during the lag phase (Figure 4, Table 1). Rpn4p levels are regulated by the 26 S proteasome via a negative feedback control mechanism [57]. It is also required for regulation of genes involved in DNA repair and other cellular processes, such as DNA damage-inducible genes *MAG1 *and *DDI1 *[33,53]. Interestingly, Rpn4p is a feedback regulator of *YAP1 *and *PDR1 *[37]. The consistent expression of *RPN4 *and its known complex functions including regulatory functions indicated a significant role of this transcription factor gene in regulating genomic adaptation networks during the lag phase. This was further demonstrated by the comparative performance of the deletion mutation response to HMF. While it was able to grow and establish a culture normally without HMF challenge, the strain harboring *Δrpn4 *failed to recover in the presence of 15 mM HMF 6 days after incubation (Figure 7A and 7B).

Although the levels of induction of *HSF1 *were not as great as *RPN4*, we found its constantly enhanced expression response to HMF was statistically significant. Up-regulated genes *HSP26 *and *SSA4 *for protein folding and refolding in this study have been reported to be regulated by Hsf1p [33,58]. It was also a positive regulator of other transcription factor genes *RPN4*, *PDR3*, *YAP5*, and *YAP6 *[32-34,36]. *HSF1 *is likely involved in the complex co-regulation networks to the HMF stress.

### Regulatory interactions of repressed genes

For 246 significantly repressed genes, we found at least 5 important regulatory genes were involved in the down-regulated expression. For example, *ARG1*, *ARG3*, *ARG4*, *ARG5,6*, *ARG7*, and *ARG8 *involved in arginine biosynthesis repressed by HMF were regulated by the transcription factor genes *ARG80 *and *ARG81*, as well as *GCN4 *(Additional file 3A and 3B). These transcription factor genes were reported to regulate arginine metabolism [59,60]. All of these genes were found to be down-regulated under the HMF stress in this study. In addition to regulation of arginine biosynthesis, *GCN4 *regulates expression of many other genes related to amino acid biosynthesis (Additional file 3B), identified by Natarajan et al [60]. Numerous genes involved in biosynthesis of histidine, leucine, and lysine were repressed under the control of *GCN4*. Among the genes repressed by HMF, a large number of genes are involved in ribosome biogenesis and protein translation processes, which were predicted to be regulated by transcription factor genes *RAP1 *and *FHL1 *(Additional file 3C). At the same time, *RAP1 *and *FHL1 *also showed repressed expression response.

### Deletion mutation response to HMF

All selective single gene deletion mutations displayed normal growth similar to their parental strain in the absence of HMF treatment on SC medium (Figure 7A). In the presence of HMF, the parental strain BY4742 showed a delayed growth response on SC medium. In contrast, all tested deletion mutations for genes *YAP1, RPN4, PDR1, PDR3, YAP4, YAP5, YAP6, ADH6, ADH7, ALD4, SNQ2, ICT1, SHP1, OTU1, MET3, MET14, CHA1, ALT1, SSA4, OYE3, NPL4, MAG1, GRE2, GRE3, ARI1, YBR062C*, and *YER137C*, displayed varied lengths of lag phase (Figure 7B). These represent growth defects at different levels in the absence of the individual genes. Among which, the most profound effect was observed by *Δrpn4 *and *Δyap1 *for transcription factor genes *RPN4 *and *YAP1 *as mentioned above. Metabolic conversion profiles were highly consistent with the growth response. As assayed by HPLC, no glucose consumption was observed for all tested strains during the lag phase (data not shown).

## Discussion

Yeast adaptation to lignocellulose derived inhibitor stress is manifest at genome level and likely during the lag phase [23]. Variation in the length of the lag phase has been widely used to measure the tolerance of strains to a specific inhibitor(s). Using DNA 70-mer long oligo microarray and qRT-PCR assays, we investigated comparative transcriptome profilings of *S. cerevisiae *during the lag phase under HMF challenge in a time-course study. Our comprehensive analyses uncovered important transcription factor genes, including *YAP1, YAP5, YAP6, PDR1, PDR3, RPN4*, and *HSF1*, as key regulators for the yeast adaptation, as well as their co-regulation and complicated regulatory networks with numerous multiple function genes. We identified more than 300 genes showing statistically significant differential expression responses that potentially affect yeast adaptation to the inhibitor challenge. Among which, more than 70 genes were consistently induced and more than 200 genes were repressed at varied stages during the lag phase. This is the first report of systematic analysis on genomic expression to inhibitor stress during the lag phase in the context of yeast adaptation. Knowledge obtained from this study provides insight into global adaptive responses of the yeast to inhibitor stress and aids the dissection of tolerance mechanisms of the yeast.

Our studies uncovered at least three significant elements for yeast adaptation to inhibitor stress and mechanisms of tolerance. The first component involves the functional enzymes and related regulatory networks directly involved in biotransformation and inhibitor detoxification. At a sublethal dose, yeasts are able to convert HMF into less toxic compound FDM. The *in situ *detoxification of HMF has been identified as a primary mechanism of the tolerance for yeast strains [5]. This is mainly accomplished via the activity of functional reductase and numerous enzymes possessing NAD(P)H-dependent aldehyde reduction activities, such as enzyme encoding genes *ADH6, ADH7, ALD4, ARI1, ARI2, ARI3, OYE3, GRE2*, and *GRE3 *[[26,27,61-64], Liu, unpublished data]. In this study, we found *ADH7*, *ARI1, GRE2*, and *ALD4 *were immediately induced by the addition of HMF, especially for *ADH7 *which displayed a greater than 30-fold increase in transcription abundance 10 min after the HMF addition and 80-fold increase at 1 h. The expression of *ADH7 *was regulated by Yap1p, Yap5p, Yap6p, and Pdr1p (Figure 6). Multiple layers of up-regulated expressions of *ADH7 *provide strong support for its extremely high levels of induction. On the other hand, it indicated the significant roles of *ADH7 *in adaptation to the aldehyde inhibitor challenge and tolerance to the inhibitor. Most reductase genes are regulated by Yap1p and related regulons Yap5p and Yap6p. A few enzyme encoding genes for example, *ALD4 *and *GRE2 *were co-regulated by Pdr1p. It should be pointed out that multiple functions of a gene are common and the co-regulation can be a reflection of the multi-functions.

**Figure 9:**
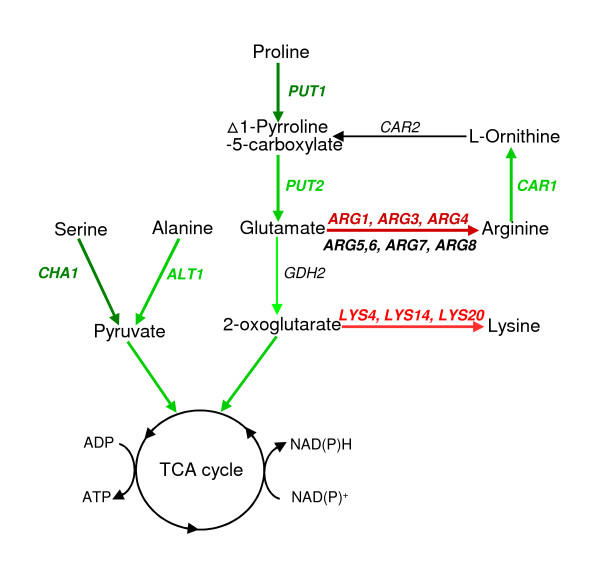
**Pathways affected toward TCA cycle**. Pathways involved in metabolisms of serine, alanine, proline, lysine, and arginine toward TCA cycle for ATP and NAD(P)H regeneration are significantly affected by HMF challenge. Bolded letters and arrowed lines indicate the levels of expressions and pathways are statistically significant. Enhanced expressions and pathways are in green and repressed in red. Black letters and arrowed lines indicate normal expressions and pathways.

As mentioned above, conversion of aldehyde inhibitors including HMF, consumes cofactor NAD(P)H and redox imbalance often causes damage in cell metabolism. We have previously demonstrated that tolerant yeast cells utilize reprogrammed pathways to detoxify aldehyde inhibitors and favored pentose phosphate pathway in regeneration of cofactors keeping a well maintained redox balance [29,65]. In this study, we found the yeast, during the lag phase, appeared to facilitate a short path to the TCA cycle from which energy and NAD(P)H regeneration can be achieved. This involved genes in the amino acids metabolism pathways closely related to the TCA cycle, both induced genes such as *CHA1*, *ALT1*, *PUT1*, *PUT2*, and *CAR1*, and repressed genes such as *ARG1*, *ARG3*, *ARG4*, *ARG5,6*, *ARG7*, *ARG8*, *LYS4*, *LYS14*, and *LYS20 *(Figure 9). The accelerated catabolism of proline, serine, and alanine, together with the reduced biosynthesis of arginine likely provided a shortcut for ATP regeneration via the TCA cycle. Thus, efficient energy metabolism can be maintained under the HMF stress. These findings suggest the altered pathway is an adaptation response that allows sufficient production of intermediate substrates for energy and NAD(P)H regeneration through the TCA cycle under the HMF challenge. Many of these genes, for example, *PUT2 *and *ALT1 *are regulated by *YAP1 *and its related YAP gene family. Yap1p has been reported as involved in the regulation of numerous other anti-oxidant genes [66-68]. It also plays a significant role for DNA damage repairing [69]. The preferred Yap1p binding site is TTACTAA [39]. We found many reductase genes that contribute to the biotransformation of the inhibitors have the Yap1p binding site in their promoter regions and are likely regulons of Yap1p.

The second element we found to be significant for yeast survival and adaptation under the HMF challenge is the PDR gene family-centered functions that are regulated by Pdr1/3p and as well as other regulator genes such as *YAP1 *and *HSF1*. Some PDR genes function as transporters of ATP-binding cassette proteins and encode plasma membrane proteins. These genes mediate membrane translocation of ions and a wide range of substrates and often exhibit multiple functions in response to a large variety of unrelated chemical stresses [42-45]. In this study, we found at least 15 members of the PDR gene family were significantly induced by HMF. The membrane and transporter activity related functions are mainly documented for these genes. For example, *TPO1 *and *TPO4 *encode proteins to function as drug/toxin transport and multidrug efflux pumps [70,71], *RSB1 *for transport ATPase, and *PDR15 *for ABC transporters, specifically. Other genes encode proteins that have multiple functions covering all of these categories, such as *SNQ2, YOR1, PDR5*, and *PDR12 *(Table 3). In addition, proteins encoded by these genes also perform functions of ATP binding and other cytoplasmic and molecular functions. Confirmed by deletion mutation assays of cell growth and qRT-PCR, we reasonably speculate that ABC transporters play a key role to export excessive HMF and endogenous toxic metabolites from intracellular environment brought about by HMF damage. As mentioned above, the shortcut of the TCA cycle could provide energy for the pumping of HMF and toxic metabolites by ABC transporters.

In this group, we observed induced transcriptional response of *RSB1 *and *ICT1*. These two genes are involved in phospholipid synthesis and transportation for membrane structure and functions, and are responsible for tolerance to organic solvents in *S. cerevisiae *[72,73]. It is possible that the induction of these PDR genes prevents the fast influx of HMF into cytoplasm and important organelles by membrane remodeling, thus, increasing the cell's tolerance to HMF. *MAG1 *encodes a 3-methyladenine (3MeA) DNA glycosylase [74], which acts in the first step of a multistage base excision repair pathway for the removal of lethal lesions such as 3MeA and protects yeast cells from killing by DNA-alkylating agents [75]. *DDI1*, located immediately upstream of *MAG1 *and transcribed in an opposite direction, encodes an ubiquitin-related protein and is involved in a DNA-damage cell-cycle checkpoint [76]. Another DNA damage related gene *RAD16 *was also induced by HMF [77]. The induction of *MAG1*, *DDI1*, and *RAD16 *in this study are consistent with the potential DNA damage by HMF and yeast defense response to the HMF challenge. Regulatory interactions of PDR gene family are complex and many genes appeared to be regulated by multiple transcription factor genes involving *PDR1, PDR3, YAP1*, and *HSF1*. Regulatory roles of *PDR1 *and *PDR3 *to HMF challenge were suggested by computational modeling [78,79]. Our deletion mutation assays using qRT-PCR suggest *PDR1 *may have direct interactive effects with more induced genes than *PDR3*, but *PGA3 *appeared to be regulated by *PDR3*. However, detailed interactions of the multiple functions of most PDR genes remain largely unknown, which can be related to the multiple regulated interactions by *YAP1, PDR1, PDR3*, and *HSF1 *as outlined by this study.

The third component of the yeast adaptation response to HMF involves degradation of damaged proteins and protein modifications mainly regulated by transcription factor genes *RPN4 *and *HSF1*. Chemical stress causes damage to protein conformation leading to protein unfolding and aggregation [80]. Small heat shock proteins, acting as chaperones, assist in folding or refolding nascent or denatured proteins and enzymes to maintain a functional conformation [81]. In this study, we found *HSP26 *and *SSA4 *encoding chaperones were significantly induced to counteract HMF stress damage to proteins. The deletion mutation of *SSA4 *displayed a significant longer lag phase under the HMF challenge, indicating its important role in adaptation and tolerance to HMF. While the presence of chaperones provides positive contribution to protein protection, severe or prolonged stress condition can result in irreversible protein damage. Misfolded or damaged proteins, especially aggregated proteins are highly toxic to cells [80]. Degradation of misfolded and damaged proteins by the ubiquitin-mediated proteasome pathway plays an important role in maintaining normal cell function and viability [80,82,83]. Denatured proteins are targeted via the covalent attachment of ubiquitin to a lysine side chain, and polyubiquitinated proteins are finally delivered to proteasome to be degraded. We observed that at least 14 ubiquitin-related and proteasome genes were induced by HMF (Figure 4B, Table 1), indicating their important functions in adaptation to the HMF stress. Strains with deletion mutations in these genes were sensitive to HMF with an extended lag phase, for example, genes *OTU1 *and *SHP1*. It was suggested that the degradation of proteins by the ubiquitin-mediated proteasome pathway has regulatory roles on cell cycle, metabolic adaptations, gene regulation, development, and differentiation [84].

As indicated by our study, many genes involved in the degradation of damaged protein and protein modifications are regulated by transcription factor gene *RPN4*. Our deletion mutation assays of *RPN4 *showed normal growth in the absence of HMF but no growth with the HMF treatment. These results confirmed the vital role of *RPN4 *involvement in adaptation to survival and coping with the HMF challenge. Since *HSF1 *is an essential gene, no deletion mutant test was performed.

## Conclusions

**Figure 10:**
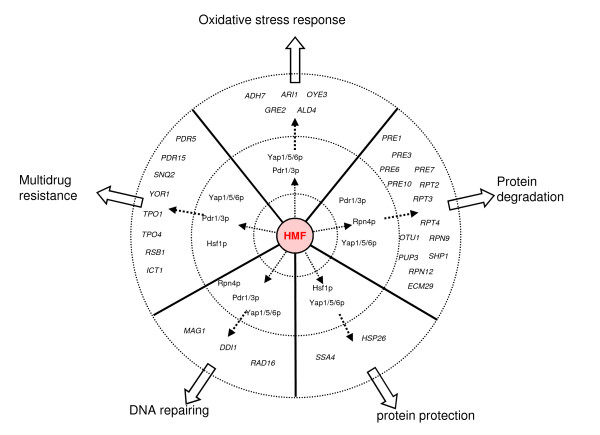
**Yeast response to HMF**. A schematic diagram of gene regulatory networks involving selective genes and significant regulatory elements in yeast response to HMF stress.

Among 365 genes identified as differentially expressed under HMF challenges, both induced and repressed genes of multiple functional categories are associated with the yeast adaptation to the inhibitor HMF during the lag phase. Transcription factor genes *YAP1*, *PDR1, PDR3, RPN4*, and *HSF1 *were identified as key regulatory genes for yeast global adaptation. Functional enzyme coding genes, for example *ARI1, ADH6*, *ADH7*, and *OYE3*, as well as gene interactions involved in the biotransformation and regulated by *YAP1*, are directly involved in the conversion of HMF into the less toxic compound FDM. PDR genes encode plasma membrane proteins and function as transporter of ATP-binding cassette proteins. The large number of induced PDR genes observed by our study suggests a hypothesis of the important PDR function of pumping HMF and endogenous toxic metabolites to maintain cell viability. Important PDR gene functions include specific transporter ATPase gene *RSB1*, toxin transporter genes *TPO1 *and *TPO4*, and multiple cellular transport facilitator genes *PDR5, PDR12, PDR15, YOR1*, and *SNQ2*. In addition, highly expressed genes involving degradation of damaged proteins and protein modifications regulated by *RPN4*, *HSF1*, and other co-regulators appear to be necessary for yeast survival and adaption to the HMF stress. Mutant strain *Δrpn4 *was unable to recover growth in the presence of HMF suggesting a significant regulatory role of *RPN4 *for many regulons. Complex gene interactions and regulatory networks as well as co-regulation events exist in response to the lignocellulose derived inhibitor HMF (Figure 10). Results from this study provide insight into mechanisms of adaptation and tolerance by the yeast *Saccharomyces cerevisiae *that will directly aid continued engineering efforts for more tolerant yeast development.

## Methods

### Strain, medium, and cultivation condition

*S. cerevisiae *strain NRRL Y-12632 (Agricultural Research Service Culture Collection, Peoria, IL, USA) was used in this study. The yeast was maintained and cultured on a synthetic complete (SC) medium as previously described [24]. Nonessential haploid *S. cerevisiae *deletion mutations generated by the Saccharomyces Genome Deletion Project [85] and the parental train BY4742 (MATα his3Δ1 leu2Δ0 lys2Δ0 ura3Δ0) were obtained from Open Biosystems (Huntsville, AL). Culture inocula were prepared using freshly grown cells harvested at logarithmic growth phase after incubation with agitation of 250 rpm at 30°C for 16 h. Cells were incubated on SC medium in a fleaker fermentation system (aerobic) at 30°C with agitation as described previously [24,28]. HMF was added into the culture at a final concentration of 30 mM 6 h after the inoculation. Cultures grown under the same conditions without the HMF treatment served as a control. Two replicated experiments were carried out for each condition.

### Cell treatment and sample collection

Cell growth was monitored by absorbance at OD_600 _during the fermentation. The time point at the HMF addition after 6-h pre-culture was designated as 0 time point. At 0, 10, 30, 60, 120 min after the HMF treatment, cell samples were harvested by centrifugation at 3645 g for 2 min at room temperature. Cell pellets were immediately frozen on dry ice and then stored at -80°C until use. Culture supernatants were taken periodically from 0 h to 54 h for metabolic profiling analysis. Glucose consumption, ethanol conversion, HMF, and FDM were measured using a high performance liquid chromatography (HPLC) system (Water Corp, Milford, MA) composed of a Waters 717 plus autosampler controlled at 10°C, Waters 590 programmable pump, an Aminex HPX-87 H column (Bio-Rad Laboratories, Hercules, CA) proceeded by a Microguard cartridge, a Spectra-Physics Spectra 100 variable wavelength UV detector (215 nm), and a Waters 2414 refractive index detector. The column was maintained at 65°C, and samples were eluted with 1.6 mM H_2_SO_4 _at 0.6 ml/min isocratic flow. A standard curve was constructed for each detected chemical and metabolic conversion product for HPLC assays as described previously [26,28].

### Microarray design and fabrication

Genome microarray of *S. cerevisiae *was fabricated with a version of 70-mer oligo set representing 6,388 genes. Using OminGrid 300 Gene Machine (Genomic Solutions, Ann Arbot, MI), a mini-array consisting of quality control genes was designed on the top of the target array for data acquisition reference during pre-scanning [86]. Replicated universal RNA controls were embedded in the target array with 32 replications for each control gene and other quality controls of DNA sequence background and slide background were included. The target genome array was printed in duplicate on a slide. Each microarray slide consisted of approximately 13,000 elements including target genes and quality controls.

### RNA isolation, probe, labeling, and hybridization

Total RNA was isolated and purified using RNeasy Mini Kit (QIAGEN, Alameda, CA, USA) using a protocol as previously described [86]. RNA integrity was verified by gel electrophoresis and NanoDrop Spectrophotometer ND-100 (NanoDrop Technologies, Inc., Wilmington, DE). RNA probe, together with incorporated RNA controls, was labeled using an indirect dUTP Cy3 or Cy5 dye as described previously [86]. Cy5 labeled RNA at 0 time point was designated as a reference and Cy3 was used to label test samples. An equal amount of at least 30 pmol Cy3 and Cy5 labeling reaction was applied for hybridization. Hybridization was performed based on Hegde et al [87] with modifications using HS 4800 Hybridization station (TECAN, Research Triangle Park, NC, USA).

### Data acquisition and analysis

Microarray slides were scanned using a GenePix 4000B scanner (Axon Instruments, Union City, CA, USA) and data acquisition was performed applying universal RNA controls using GenPix Pro V 6.0 software (Molecular Devices, Sunnyvale, CA, USA). A pre-scan control mini-array was used to adjust PMT Gain against Cy3 and Cy5 channels and the ratios of signal intensities between Cy3 and Cy5 were balanced to 1.0 using the calibration control as described previously [86]. Each spot was individually examined and adjusted or flagged out if necessary. Microarray data was deposited at the Gene Expression Omnibus (GEO) database under Accession "GSE22939". Median of foreground signal intensity subtracted by background for each dye channel was used for analysis. Raw data for each slide were normalized based on spike-in control gene *CAB*, and normalized data were analyzed using GeneSpring GX 10.0 (Agilent Technologies, Santa Clara, CA, USA). Briefly, expression values less than 100 in 7 of 16 samples were filtered out from probesets, then a 2-way ANOVA analysis (p ≤ 0.05) was performed. Genes showing statistically significant differential expressions with a minimum of 2-fold changes were selected for Principal Component Analysis and clustering analysis by Hierarchical and Self Organizing Maps. Interaction pathway analyses were modified and incorporated with the most up-to-date information. Gene functions were annotated using the *Saccharomyces *genome database (SGD) Gene Ontology Term Finder version 0.83 http://www.yeastgenome.org/cgi-bin/GO/goTermFinder.pl[88] and MIPS Functional Catalogue [89] with a significant cut-off value of p < 0.01. Transcription factor analysis was performed using the T-Profiler tool [30], and the selected transcription factors were further analyzed using YEASTRACT [31].

### Deletion mutation response to HMF

Twenty-seven single gene deletion mutations from Saccharomyces Genome Deletion Sets were selected for growth response to HMF. These genes include available non essential genes and transcription factor genes *YAP1, RPN4, PDR1, PDR3, YAP4, YAP5, YAP6, ADH6, ADH7, ALD4, SNQ2, ICT1, SHP1, OTU1, MET3, MET14, CHA1, ALT1, SSA4, OYE3, NPL4, MAG1, GRE2, GRE3, ARI1, YBR062C*, and *YER137C*. A parental strain BY4742 (WT) grown with and without HMF treatment served as a control. Each tested strain was grown on a 4 ml SC medium in a 15-ml tube at 30°C with agitation of 250 rpm. Culture inocula were prepared using freshly grown cells harvested at logarithmic growth phase after incubation for 16 h. The initial OD at 600 nm of the inoculated medium for each deletion strain culture was adjusted to the same level and inoculated onto the SC medium with a final HMF concentration of 15 mM. Cell growth was monitored by absorbance at OD_600 _and culture supernatants were taken periodically for HPLC analysis of glucose consumption, ethanol production, HMF, and FDM conversion as described above.

### Quantitative real time RT-PCR assays

Regulatory interactions among induced expression by transcription factor gene *PDR1 *and *PDR3 *were verified applying a single gene deletion mutation *Δpdr1 *and *Δpdr3 *from Saccharomyces Genome Deletion Set using qRT-PCR. Primer design (Additional file 5), PCR profiles, and assay method are as previously described [29,90]. HMF and furfural were added into the medium at a final concentration of 15 mM each after 6 h pre-culture. The time point at the addition of inhibitors was designated as 0 h. Cell samples were harvested at 0 and 2 h during the lag phase and RNA extracted as previously described [86].

## List of abbreviations

ABC: ATP-binding cassette; ChIP-chip: chromatin immunoprecipitation associated with microarrays; FDM: furan-2, 5-dimethanol; GEO: Gene Expression Omnibus; GO: gene ontology; HMF: 5-hydroxymethylfurfural; HPLC: high performance liquid chromatography; PACE: proteasome-associated control elements; PCA: Principal Component Analysis; PDRE: pleiotropic drug response elements; ROS: reactive oxygen species; SC medium: synthetic complete medium; SNP: single nucleotide polymorphism; SGD: *Saccharomyces *genome database; SOM: Self Organizing Maps; TCA cycle: tricarboxylic acid cycle; TF: transcription factor; YEASTRACT: Yeast Search for Transcriptional Regulators And Consensus Tracking; YRE: Yap1p response elements.

## Authors' contributions

ZLL conceived the project, designed and fabricated yeast DNA microarray, and conducted the microarray experiments. MM performed the qRT-PCR and deletion mutation assays. MM and ZLL analyzed data and wrote the manuscript. All authors read and approved the final manuscript.

## Supplementary Material

Additional file 1 **Differential expression of significantly affected genes of *Saccharomyces cerevisiae *in response to HMF challenges during the lag phase**. Click here for file

Additional file 2 **Gene Ontology (GO) categories and terms for significantly repressed genes by HMF (30 mM) challenge in *Saccharomyces cerevisiae *NRRL Y-12632**. Click here for file

Additional file 3 **Regulatory interaction networks for repressed genes under HMF stress**. Click here for file

Additional file 4 **Protein binding motifs and binding elements for significantly induced genes by HMF challenge during the lag phase in *Saccharomyces cerevisiae***. Click here for file

Additional file 5 **Primers used for mRNA expression analysis for *Saccharomyces cerevisiae *by real-time qRT-PCR using SYBR Green I**. Click here for file
