# Supplementary material for: Comparative transcriptome profiling analyses during the lag phase uncover YAP1, PDR1, PDR3, RPN4, and HSF1 as key regulatory genes in genomic adaptation to the lignocellulose derived inhibitor HMF for Saccharomyces cerevisiae
Source: BMC Genomics. 2010 Nov 24;11:660. doi: 10.1186/1471-2164-11-660 (PMC3091778; doi:10.1186/1471-2164-11-660)
Supplement: Additional file 1 — Differential expression of significantly affected genes of Saccharomyces cerevisiae in response to HMF challenges during the lag phase. [file 1471-2164-11-660-S1.DOC]

Additional file 1. Differential expressions of significantly affected genes in *Saccharomyces cerevisiae* in response to HMF challenges during the lag phase

| NO. | Systematic Name | Standard Name | Fold change | | | | | | | |
| --- | --- | --- | --- | --- | --- | --- | --- | --- | --- | --- |
|  |  |  | HMF | | | | Control | | | |
|  |  |  | 0.2 h | 0.5 h | 1 h | 2 h | 0.2 h | 0.5 h | 1 h | 2 h |
| 1 | YAR073W | *IMD1* | +2.2 | +2.1 | +3.4 | +2.7 | +1.5 | +1.3 | +1.0 | -1.1 |
| 2 | YBL041W | *PRE7* | +3.5 | +2.6 | +2.6 | +1.9 | +2.0 | +1.5 | +1.1 | +1.5 |
| 3 | YBL058W | *SHP1* | +2.5 | +2.0 | +2.1 | +2.8 | +1.8 | +1.3 | -1.0 | +1.3 |
| 4 | YBL078C | *ATG8* | +4.5 | +3.3 | +2.7 | +3.5 | +2.5 | +1.6 | +1.0 | +1.6 |
| 5 | YBL101W-A | *YBL101W-A* | +1.4 | +1.6 | +2.6 | +6.4 | +1.3 | +1.1 | -1.0 | +2.3 |
| 6 | YBR062C | *YBR062C* | +2.5 | +2.4 | +2.4 | +2.1 | +1.9 | +1.3 | -1.0 | +1.3 |
| 7 | YBR072W | *HSP26* | +10.8 | +1.8 | +2.0 | +8.0 | +3.5 | +2.6 | +1.2 | +1.8 |
| 8 | YBR114W | *RAD16* | +2.8 | +2.1 | +2.8 | +1.7 | +2.2 | +1.6 | +1.2 | +1.1 |
| 9 | YBR170C | *NPL4* | +2.4 | +2.7 | +2.2 | +1.5 | +1.9 | +1.4 | +1.1 | -1.2 |
| 10 | YBR255C-A | *YBR255C-A* | +4.0 | +2.0 | +1.8 | +2.1 | +1.8 | +1.3 | +1.0 | +1.3 |
| 11 | YCL064C | *CHA1* | +13.3 | +17.7 | +15.8 | +7.3 | +1.9 | +1.3 | +1.5 | +2.0 |
| 12 | YDL007W | *RPT2* | +3.3 | +2.6 | +2.6 | +2.4 | +2.0 | +1.6 | +1.0 | +1.2 |
| 13 | YDL020C | *RPN4* | +1.9 | +2.5 | +2.8 | +3.3 | +2.7 | +1.8 | +1.0 | +1.4 |
| 14 | YDL021W | *GPM2* | +3.5 | +1.6 | +1.7 | +2.8 | +2.5 | +1.7 | -1.2 | +1.3 |
| 15 | YDR011W | *SNQ2* | +4.7 | +3.8 | +6.4 | +4.1 | +2.3 | +1.3 | +1.3 | +1.1 |
| 16 | YDR210W-B | *YDR210W-B* | +1.9 | +1.7 | +2.6 | +4.4 | +1.7 | +1.3 | +1.1 | +1.7 |
| 17 | YDR316W-B | *YDR316W-B* | +1.8 | +1.8 | +2.6 | +3.2 | +1.6 | +1.3 | -1.0 | +1.7 |
| 18 | YDR365W-B | *YDR365W-B* | +1.7 | +1.7 | +2.2 | +4.3 | +1.6 | +1.2 | +1.0 | +1.5 |
| 19 | YDR394W | *RPT3* | +2.9 | +2.0 | +2.5 | +2.5 | +1.9 | +1.4 | +1.1 | +1.2 |
| 20 | YDR406W | *PDR15* | +9.5 | +3.5 | +5.4 | +7.3 | +3.1 | +1.7 | +1.5 | +1.5 |
| 21 | YDR427W | *RPN9* | +2.7 | +2.4 | +2.4 | +2.1 | +2.0 | +1.4 | -1.1 | +1.1 |
| 22 | YDR515W | *SLF1* | +2.7 | +2.7 | +2.4 | +2.7 | +1.7 | +1.3 | -1.0 | +2.0 |
| 23 | YER012W | *PRE1* | +2.7 | +2.4 | +2.1 | +1.8 | +1.6 | +1.1 | -1.2 | +1.0 |
| 24 | YER094C | *PUP3* | +2.4 | +2.2 | +2.9 | +2.1 | +1.8 | +1.3 | +1.0 | +1.2 |
| 25 | YER103W | *SSA4* | +13.2 | +1.5 | +2.0 | +2.6 | +2.9 | +1.7 | +1.1 | +1.3 |
| 26 | YER142C | *MAG1* | +4.3 | +4.5 | +5.1 | +3.7 | +2.3 | +1.4 | +1.4 | +1.7 |
| 27 | YER143W | *DDI1* | +2.4 | +2.2 | +2.4 | +1.7 | +1.5 | +1.1 | +1.2 | +1.2 |
| 28 | YFL044C | *OTU1* | +2.3 | +1.9 | +3.3 | +2.3 | +1.6 | +1.2 | -1.2 | -1.4 |
| 29 | YFR052W | *RPN12* | +2.8 | +2.4 | +2.6 | +2.5 | +2.1 | +1.8 | +1.0 | +1.7 |
| 30 | YGL062W | *PYC1* | +2.3 | +2.6 | +3.1 | +2.1 | +2.2 | +1.3 | +1.1 | +1.3 |
| 31 | YGL157W | *ARI1* | +12.3 | +21.3 | +29.2 | +27.2 | +1.7 | +1.0 | +1.1 | -1.2 |
| 32 | YGR035C | *YGR035C* | +3.1 | +3.8 | +4.1 | +5.9 | +1.4 | +1.2 | +1.1 | +2.2 |
| 33 | YGR111W | *YGR111W* | +2.1 | +2.2 | +4.3 | +7.1 | +2.6 | +1.8 | +1.4 | +1.2 |
| 34 | YGR281W | *YOR1* | +4.7 | +3.0 | +4.4 | +3.5 | +2.4 | +1.5 | +1.6 | +1.6 |
| 35 | YHL030W | *ECM29* | +4.6 | +3.3 | +3.6 | +2.6 | +2.1 | +1.4 | +1.1 | +1.4 |
| 36 | YHR037W | *PUT2* | +3.7 | +3.7 | +4.7 | +3.3 | +2.2 | +1.5 | +1.3 | +1.1 |
| 37 | YHR138C | *YHR138C* | +4.2 | +2.8 | +2.4 | +2.8 | +2.5 | +1.7 | +1.1 | +2.2 |
| 38 | YJL001W | *PRE3* | +3.0 | +3.0 | +2.6 | +2.1 | +2.0 | +1.5 | +1.1 | +1.2 |
| 39 | YJR010W | *MET3* | +2.5 | +2.7 | +3.3 | +3.6 | +1.8 | +1.5 | +1.3 | +1.1 |
| 40 | YKL001C | *MET14* | +1.7 | +2.6 | +2.8 | +3.2 | +1.3 | +1.3 | -1.1 | +1.1 |
| 41 | YKR011C | *YKR011C* | +4.6 | +2.3 | +1.9 | +1.3 | +2.5 | +1.7 | +1.2 | +1.2 |
| 42 | YLL028W | *TPO1* | +4.5 | +3.3 | +5.1 | +4.3 | +1.8 | +1.4 | +1.1 | +1.3 |
| 43 | YLL056C | *YLL056C* | +2.0 | +2.0 | +5.8 | +6.2 | +2.2 | +1.5 | +1.3 | +1.2 |
| 44 | YLR089C | *ALT1* | +2.9 | +3.1 | +4.1 | +4.9 | +2.1 | +1.5 | +1.3 | +1.4 |
| 45 | YLR099C | *ICT1* | +3.4 | +2.9 | +4.8 | +6.2 | +2.0 | +1.4 | +1.3 | +1.8 |
| 46 | YML007W | *YAP1* | +3.3 | +2.1 | +2.5 | +2.2 | +2.0 | +1.2 | +1.1 | +1.1 |
| 47 | YML125C | *PGA3* | +1.6 | +2.7 | +3.0 | +2.4 | +1.2 | +1.0 | -1.3 | +1.1 |
| 48 | YML130C | *ERO1* | +2.3 | +2.4 | +2.8 | +2.3 | +1.6 | +1.3 | -1.2 | +1.1 |
| 49 | YNL155W | *YNL155W* | +3.7 | +2.4 | +2.2 | +1.9 | +1.8 | +1.1 | -1.0 | +1.5 |
| 50 | YOL038W | *PRE6* | +2.0 | +2.2 | +2.8 | +2.3 | +1.6 | +1.2 | -1.1 | +1.4 |
| 51 | YOL151W | *GRE2* | +8.0 | +7.6 | +10.4 | +12.0 | +1.9 | +1.6 | +1.1 | +1.2 |
| 52 | YOR007C | *SGT2* | +2.8 | +2.5 | +2.9 | +3.0 | +2.1 | +1.4 | +1.1 | +1.4 |
| 53 | YOR009W | *TIR4* | -1.3 | +1.3 | +4.6 | +5.9 | +1.1 | +1.2 | -1.2 | -2.0 |
| 54 | YOR052C | *YOR052C* | +4.1 | +2.6 | +1.8 | +2.4 | +2.0 | +1.4 | -1.2 | +1.2 |
| 55 | YOR059C | *YOR059C* | +2.1 | +2.6 | +2.2 | +2.2 | +2.2 | +1.6 | +1.1 | +1.2 |
| 56 | YOR153W | *PDR5* | +30.0 | +19.0 | +30.6 | +23.7 | +2.3 | +1.4 | +1.4 | +1.5 |
| 57 | YOR259C | *RPT4* | +3.1 | +2.6 | +3.2 | +2.5 | +1.9 | +1.3 | +1.2 | +1.5 |
| 58 | YOR273C | *TPO4* | +2.7 | +2.0 | +3.0 | +2.6 | +1.6 | +1.1 | +1.1 | +1.5 |
| 59 | YOR306C | *MCH5* | +2.6 | +4.9 | +7.8 | +7.2 | +2.4 | +1.8 | +1.5 | +1.1 |
| 60 | YOR362C | *PRE10* | +2.4 | +2.2 | +2.2 | +1.9 | +1.9 | +1.3 | +1.0 | +1.2 |
| 61 | YOR374W | *ALD4* | +4.3 | +3.2 | +2.7 | +3.2 | +3.6 | +1.8 | +1.5 | +1.8 |
| 62 | YPL058C | *PDR12* | +7.1 | +4.1 | +6.2 | +3.3 | +2.0 | +1.3 | +1.7 | -1.1 |
| 63 | YPL111W | *CAR1* | +3.4 | +2.2 | +1.9 | +1.6 | +1.7 | -1.1 | -1.1 | -1.0 |
| 64 | YBL107C | *YBL107C* | +2.4 | +2.6 | +2.1 | +2.9 | +1.7 | +1.5 | -1.1 | +2.0 |
| 65 | YCR105W | *ADH7* | +38.4 | +60.3 | +81.3 | +39.5 | -2.9 | -1.3 | -2.2 | +1.3 |
| 66 | YDR034W-B | *YDR034W-B* | +7.2 | +5.0 | +3.6 | +4.5 | +4.5 | +3.0 | +1.8 | +1.8 |
| 67 | YER137C | *YER137C* | +2.9 | +2.4 | +2.5 | +3.6 | +2.0 | +1.7 | -1.0 | +2.2 |
| 68 | YLR142W | *PUT1* | +4.8 | +6.9 | +10.8 | +43.0 | +1.4 | +3.5 | -1.3 | +5.5 |
| 69 | YOR049C | *RSB1* | +15.7 | +6.9 | +9.5 | +8.6 | +4.4 | +1.9 | +1.6 | +1.1 |
| 70 | YPL156C | *PRM4* | +3.4 | +2.8 | +3.5 | +3.5 | +1.9 | +1.5 | +1.2 | -1.3 |
| 71 | YPL171C | *OYE3* | +5.9 | +3.9 | +5.3 | +17.7 | +2.2 | +2.0 | -1.2 | +1.3 |
| 72 | Q0050 | *AI1* | +1.1 | -1.1 | -1.1 | +1.5 | +2.5 | +1.7 | +1.3 | +4.2 |
| 73 | Q0110 | *BI2* | +1.3 | +1.0 | -1.2 | +1.2 | +2.3 | +1.8 | +1.7 | +4.4 |
| 74 | Q0115 | *BI3* | -1.1 | -1.4 | -1.3 | +1.3 | +2.0 | +1.5 | +1.5 | +4.2 |
| 75 | YBL015W | *ACH1* | +1.3 | -1.4 | -1.4 | -1.3 | +2.3 | +1.9 | +1.4 | +1.4 |
| 76 | YBR035C | *PDX3* | +1.1 | -1.4 | -1.3 | -1.1 | +2.0 | +1.4 | -1.1 | +1.4 |
| 77 | YBR047W | *FMP23* | +1.0 | +1.3 | +1.1 | +1.1 | +2.2 | +1.7 | -1.0 | +1.7 |
| 78 | YBR256C | *RIB5* | +1.1 | -1.1 | -1.1 | -1.1 | +2.5 | +1.9 | +1.4 | +1.9 |
| 79 | YBR298C | *MAL31* | +1.5 | -2.1 | -1.4 | -1.3 | +2.8 | +1.9 | +1.7 | +1.4 |
| 80 | YCR099C | *YCR099C* | +1.6 | -1.2 | -1.4 | -1.4 | +3.2 | +1.8 | +1.2 | +1.0 |
| 81 | YCR100C | *YCR100C* | +1.4 | -1.2 | -1.5 | -1.3 | +2.7 | +1.6 | +1.0 | -1.2 |
| 82 | YDL085W | *NDE2* | +1.1 | -1.1 | -1.1 | +1.1 | +2.5 | +1.9 | +1.6 | +1.3 |
| 83 | YDR519W | *FPR2* | -1.4 | -1.1 | +1.3 | +1.1 | +3.0 | +1.3 | +1.2 | +1.3 |
| 84 | YER024W | *YAT2* | -1.1 | -1.5 | -1.1 | -1.2 | +2.5 | +1.8 | +1.4 | +1.6 |
| 85 | YER059W | *PCL6* | +1.1 | -1.1 | -1.0 | -1.3 | +2.1 | +1.4 | +1.2 | +1.4 |
| 86 | YGR021W | *HAH1* | -1.2 | -1.1 | -1.3 | -1.1 | +1.4 | +1.3 | -1.3 | +3.4 |
| 87 | YGR289C | *MAL11* | +1.5 | -1.6 | +1.0 | +1.0 | +3.3 | +2.6 | +1.8 | +1.3 |
| 88 | YHR029C | *YHI9* | -1.1 | -1.5 | -1.0 | +1.0 | +2.2 | +1.4 | +1.3 | +1.6 |
| 89 | YHR047C | *AAP1* | -1.0 | -1.3 | +1.0 | +1.0 | +2.5 | +1.6 | +1.3 | +1.2 |
| 90 | YKL023W | *YKL023W* | -1.2 | -1.2 | -1.1 | +1.2 | +2.3 | +1.4 | +1.0 | +1.3 |
| 91 | YLR290C | *YLR290C* | +1.4 | -1.1 | -1.2 | +1.2 | +2.5 | +2.0 | +1.1 | +1.6 |
| 92 | YLR327C | *TMA10* | +1.1 | +1.1 | -1.4 | +1.1 | +2.1 | +1.7 | +1.1 | +2.9 |
| 93 | YMR096W | *SNZ1* | -1.5 | -1.3 | +1.0 | +1.2 | +2.4 | +1.5 | +1.1 | +1.3 |
| 94 | YMR194C-A | *YMR194C-A* | +1.0 | -1.5 | -1.0 | -1.2 | +2.1 | +1.2 | -1.1 | +1.2 |
| 95 | YMR251W-A | *HOR7* | +1.3 | -1.1 | -1.3 | -1.0 | +2.2 | +1.6 | +1.3 | +1.9 |
| 96 | YMR320W | *YMR320W* | +2.6 | -1.4 | -1.5 | -1.9 | +2.7 | +1.4 | -1.1 | -1.4 |
| 97 | YNR034W | *SOL1* | +1.2 | +1.0 | -1.1 | -1.5 | +2.1 | +1.5 | +1.3 | -1.1 |
| 98 | YOL117W | *RRI2* | +1.1 | -1.1 | -1.0 | +1.1 | +2.5 | +1.8 | +1.1 | -1.0 |
| 99 | YPL219W | *PCL8* | +1.2 | -1.2 | -1.1 | -1.2 | +2.4 | +1.6 | +1.0 | -1.3 |
| 100 | YLR312C | *YLR312C* | +1.8 | +1.0 | +1.2 | +1.3 | +4.6 | +2.6 | +2.1 | +1.7 |
| 101 | YBL069W | *AST1* | -2.0 | -2.0 | -1.3 | -1.3 | +2.0 | +1.5 | -1.1 | -1.2 |
| 102 | YBL072C | *RPS8A* | -1.6 | -2.4 | -1.6 | -1.3 | +1.6 | +1.2 | -1.1 | +1.3 |
| 103 | YBL087C | *RPL23A* | -1.7 | -2.4 | -1.5 | -1.3 | +1.6 | +1.1 | +1.0 | +1.2 |
| 104 | YBR084C-A | *RPL19A* | -1.8 | -2.4 | -1.6 | -1.4 | +1.4 | +1.2 | -1.2 | +1.4 |
| 105 | YBR117C | *TKL2* | -1.1 | -2.9 | -2.0 | -1.5 | +2.5 | +1.9 | +1.8 | +1.1 |
| 106 | YBR147W | *RTC2* | -1.6 | -2.2 | -3.1 | -3.1 | +3.8 | +2.3 | +1.6 | +1.7 |
| 107 | YBR181C | *RPS6B* | -1.6 | -2.3 | -1.4 | -1.4 | +1.8 | +1.3 | +1.1 | +1.4 |
| 108 | YBR189W | *RPS9B* | -1.6 | -2.4 | -1.6 | -1.6 | +1.4 | +1.2 | -1.1 | -1.1 |
| 109 | YBR218C | *PYC2* | -1.1 | -1.8 | -1.6 | -1.7 | +2.5 | +1.6 | +1.3 | +1.3 |
| 110 | YBR299W | *MAL32* | -1.4 | -2.5 | -2.1 | -2.0 | +2.6 | +1.9 | +1.6 | +1.2 |
| 111 | YBR300C | *YBR300C* | -1.1 | -2.4 | -1.5 | -1.3 | +2.4 | +1.8 | +1.5 | +1.3 |
| 112 | YCL009C | *ILV6* | -1.4 | -2.2 | -1.6 | -1.6 | +1.4 | +1.3 | -1.1 | +1.2 |
| 113 | YDL055C | *PSA1* | -1.8 | -2.0 | -1.6 | -1.5 | +1.5 | +1.2 | -1.0 | +1.1 |
| 114 | YDL082W | *RPL13A* | -1.5 | -2.4 | -1.5 | -1.3 | +1.6 | +1.4 | -1.0 | +1.1 |
| 115 | YDL083C | *RPS16B* | -1.8 | -2.2 | -1.7 | -1.4 | +1.8 | +1.3 | -1.0 | +1.2 |
| 116 | YDL174C | *DLD1* | +1.4 | -1.5 | -1.8 | -2.2 | +2.0 | +1.4 | -1.1 | -1.1 |
| 117 | YDL198C | *GGC1* | -1.8 | -2.3 | -1.7 | -2.3 | +1.9 | +1.6 | +1.5 | +1.2 |
| 118 | YDL222C | *FMP45* | -1.0 | -2.4 | -2.7 | -1.8 | +2.8 | +1.9 | +1.3 | +1.6 |
| 119 | YDL229W | *SSB1* | -2.0 | -2.4 | -1.3 | -1.4 | +1.6 | +1.2 | +1.0 | +1.2 |
| 120 | YDR064W | *RPS13* | -1.7 | -2.2 | -1.6 | -1.6 | +1.5 | +1.3 | -1.1 | +1.1 |
| 121 | YDR158W | *HOM2* | -1.8 | -3.0 | -1.9 | -2.3 | +1.8 | +1.4 | +1.2 | +1.3 |
| 122 | YDR354W | *TRP4* | -2.0 | -1.9 | -1.4 | -1.7 | +1.7 | +1.4 | -1.0 | +1.0 |
| 123 | YDR382W | *RPP2B* | -1.3 | -2.2 | -1.6 | -1.3 | +1.8 | +1.4 | +1.1 | +1.2 |
| 124 | YDR385W | *EFT2* | -1.6 | -2.2 | -1.3 | -1.3 | +1.9 | +1.4 | +1.2 | +1.0 |
| 125 | YDR418W | *RPL12B* | -2.2 | -2.7 | -1.5 | -1.2 | +1.7 | +1.4 | +1.1 | +1.2 |
| 126 | YDR450W | *RPS18A* | -1.6 | -2.2 | -1.6 | -1.7 | +1.6 | +1.3 | -1.1 | -1.1 |
| 127 | YEL054C | *RPL12A* | -2.0 | -2.5 | -1.3 | -1.3 | +1.7 | +1.5 | +1.2 | +1.4 |
| 128 | YER074W | *RPS24A* | -2.1 | -2.3 | -1.7 | -1.3 | +1.5 | +1.3 | -1.1 | +1.3 |
| 129 | YER081W | *SER3* | +1.3 | -2.7 | -2.2 | -2.0 | +1.6 | +1.4 | -1.2 | -1.1 |
| 130 | YER117W | *RPL23B* | -1.8 | -2.3 | -1.5 | -1.4 | +1.5 | +1.2 | -1.0 | +1.2 |
| 131 | YER187W-A | *YER187W-A* | -1.2 | -2.0 | -1.5 | -1.4 | +2.7 | +1.9 | -1.3 | -1.6 |
| 132 | YFR023W | *PES4* | -1.4 | -1.6 | -2.0 | -1.5 | +2.3 | -1.4 | -1.1 | -1.1 |
| 133 | YFR031C-A | *RPL2A* | -1.9 | -3.3 | -1.6 | -1.4 | +1.5 | +1.4 | +1.1 | +1.1 |
| 134 | YGL030W | *RPL30* | -1.6 | -2.0 | -1.7 | -1.7 | +1.5 | +1.2 | -1.1 | +1.0 |
| 135 | YGL031C | *RPL24A* | -1.9 | -2.2 | -1.5 | -1.4 | +1.5 | +1.2 | -1.1 | +1.5 |
| 136 | YGL076C | *RPL7A* | -1.8 | -3.1 | -1.8 | -1.4 | +1.6 | +1.3 | -1.1 | +1.1 |
| 137 | YGL123W | *RPS2* | -1.7 | -2.9 | -1.6 | -1.4 | +1.5 | +1.3 | -1.0 | +1.2 |
| 138 | YGL125W | *MET13* | -1.8 | -2.9 | -1.6 | -1.2 | +2.1 | +1.9 | +1.5 | +2.0 |
| 139 | YGL135W | *RPL1B* | -1.9 | -2.4 | -1.4 | -1.6 | +1.6 | +1.3 | +1.1 | +1.0 |
| 140 | YGL148W | *ARO2* | -1.5 | -2.3 | -1.8 | -1.5 | +1.8 | +1.3 | -1.0 | +1.1 |
| 141 | YGL149W | *YGL149W* | -1.5 | -2.1 | -1.5 | -1.4 | +1.8 | +1.3 | -1.0 | +1.2 |
| 142 | YGL202W | *ARO8* | -1.4 | -2.2 | -1.3 | -1.4 | +2.1 | +1.3 | +1.2 | +1.3 |
| 143 | YGL230C | *YGL230C* | -1.7 | -2.0 | -1.6 | -1.7 | +1.6 | +1.2 | -1.2 | +1.0 |
| 144 | YGR138C | *TPO2* | -1.1 | -2.3 | -1.5 | -1.2 | +2.6 | +2.3 | +1.3 | +1.0 |
| 145 | YGR286C | *BIO2* | -2.2 | -1.7 | -1.4 | -1.6 | +1.6 | +1.3 | +1.0 | +1.5 |
| 146 | YGR291C | *YGR291C* | -1.7 | -3.5 | -1.4 | -1.5 | +1.8 | +1.6 | +1.2 | -1.1 |
| 147 | YGR292W | *MAL12* | -1.8 | -2.6 | -2.4 | -2.1 | +2.2 | +1.6 | +1.2 | -1.0 |
| 148 | YHL016C | *DUR3* | +1.8 | -2.7 | -1.7 | -2.3 | +2.6 | +1.6 | +1.9 | +2.3 |
| 149 | YHR018C | *ARG4* | -2.7 | -2.4 | -2.1 | -2.0 | +2.7 | +1.6 | +1.6 | +1.7 |
| 150 | YHR071W | *PCL5* | -1.5 | -1.7 | -1.8 | -2.8 | +2.4 | +1.8 | +1.4 | +2.7 |
| 151 | YHR203C | *RPS4B* | -1.6 | -2.4 | -1.6 | -1.5 | +1.6 | +1.3 | -1.0 | +1.2 |
| 152 | YIL059C | *YIL059C* | +2.1 | -1.4 | -3.4 | -2.4 | +3.2 | +2.4 | -1.0 | +1.2 |
| 153 | YIL116W | *HIS5* | -1.5 | -2.0 | -1.7 | -2.1 | +2.1 | +1.5 | +1.2 | +1.4 |
| 154 | YIL164C | *NIT1* | -1.1 | -2.1 | -1.7 | -1.8 | +2.6 | +1.9 | +1.8 | +2.2 |
| 155 | YIL165C | *YIL165C* | -1.2 | -2.7 | -1.9 | -2.2 | +2.9 | +1.7 | +1.5 | +1.8 |
| 156 | YJL056C | *ZAP1* | -2.0 | -1.4 | -1.6 | -2.3 | +1.6 | +1.6 | -1.0 | +1.0 |
| 157 | YJL220W | *YJL220W* | -1.5 | -1.7 | -3.9 | -1.3 | +3.1 | +1.5 | +1.5 | -1.0 |
| 158 | YJR016C | *ILV3* | -2.5 | -2.5 | -1.5 | -1.5 | +1.9 | +1.5 | +1.2 | +1.3 |
| 159 | YJR025C | *BNA1* | -1.2 | -1.9 | -2.1 | -2.3 | +1.9 | +1.5 | +1.2 | +1.9 |
| 160 | YJR130C | *STR2* | -1.5 | -1.8 | -1.4 | -1.9 | +2.3 | +1.6 | +1.3 | +1.3 |
| 161 | YKR050W | *TRK2* | -1.5 | -2.2 | -1.3 | -1.4 | +1.9 | +1.3 | +1.0 | -1.2 |
| 162 | YLR112W | *YLR112W* | -1.5 | -2.6 | -1.5 | -1.4 | +1.4 | +1.2 | +1.2 | +1.0 |
| 163 | YLR152C | *YLR152C* | -1.5 | -2.8 | -2.5 | -1.6 | +2.7 | +1.6 | +1.2 | -1.1 |
| 164 | YLR167W | *RPS31* | -1.6 | -2.1 | -1.8 | -1.5 | +1.5 | +1.1 | -1.2 | -1.1 |
| 165 | YLR344W | *RPL26A* | -1.8 | -2.0 | -1.5 | -1.5 | +1.4 | +1.2 | -1.1 | +1.1 |
| 166 | YLR448W | *RPL6B* | -2.0 | -1.9 | -1.4 | -1.4 | -1.5 | +1.2 | -1.0 | +1.2 |
| 167 | YML024W | *RPS17A* | -1.6 | -2.1 | -1.5 | -1.2 | +1.7 | +1.3 | -1.0 | +1.5 |
| 168 | YML026C | *RPS18B* | -1.6 | -2.3 | -1.7 | -1.6 | +1.6 | +1.3 | -1.1 | +1.1 |
| 169 | YML063W | *RPS1B* | -1.7 | -2.2 | -1.5 | -1.4 | +1.8 | +1.3 | +1.1 | +1.2 |
| 170 | YMR011W | *HXT2* | -1.7 | -1.5 | -1.8 | -3.0 | +2.7 | +1.4 | +1.6 | +1.4 |
| 171 | YMR143W | *RPS16A* | -1.7 | -2.6 | -1.4 | -1.5 | +1.7 | +1.4 | +1.1 | +1.3 |
| 172 | YMR315W | *YMR315W* | -1.2 | -2.2 | -1.8 | -1.2 | +2.0 | +1.3 | -1.0 | +1.1 |
| 173 | YNL031C | *HHT2* | -1.7 | -2.2 | -1.5 | -1.1 | +1.4 | +1.2 | -1.1 | +1.3 |
| 174 | YNL067W | *RPL9B* | -1.6 | -2.6 | -1.8 | -1.5 | +1.6 | +1.2 | -1.0 | +1.2 |
| 175 | YNL069C | *RPL16B* | -2.1 | -2.5 | -1.4 | -1.4 | +1.6 | +1.1 | +1.2 | +1.2 |
| 176 | YNL104C | *LEU4* | -2.0 | -2.8 | -1.3 | -1.8 | +1.7 | +1.3 | +1.1 | +1.3 |
| 177 | YNL209W | *SSB2* | -1.9 | -2.3 | -1.2 | -1.4 | +1.6 | +1.3 | +1.1 | +1.1 |
| 178 | YNL301C | *RPL18B* | -1.6 | -2.7 | -1.4 | -1.3 | +1.8 | +1.4 | +1.2 | +1.1 |
| 179 | YOL120C | *RPL18A* | -1.7 | -2.8 | -1.5 | -1.7 | +1.6 | +1.2 | +1.0 | +1.1 |
| 180 | YOR063W | *RPL3* | -1.7 | -2.3 | -1.3 | -1.7 | +1.7 | +1.2 | +1.1 | -1.1 |
| 181 | YOR096W | *RPS7A* | -1.8 | -2.5 | -1.4 | -1.4 | +1.6 | +1.2 | +1.1 | +1.3 |
| 182 | YOR133W | *EFT1* | -1.7 | -2.4 | -1.4 | -1.8 | +1.7 | +1.2 | +1.1 | -1.2 |
| 183 | YOR161C | *PNS1* | -1.8 | -2.9 | -2.7 | -3.2 | +3.0 | +1.7 | +1.5 | -1.3 |
| 184 | YPL079W | *RPL21B* | -1.7 | -2.5 | -1.4 | -1.3 | +1.6 | +1.3 | +1.1 | +1.4 |
| 185 | YPL081W | *RPS9A* | -1.4 | -2.3 | -1.5 | -1.3 | +1.6 | +1.3 | +1.0 | +1.4 |
| 186 | YPL090C | *RPS6A* | -1.7 | -2.4 | -1.4 | -1.5 | +1.8 | +1.3 | +1.0 | +1.3 |
| 187 | YPL220W | *RPL1A* | -1.9 | -2.4 | -1.4 | -1.7 | +1.6 | +1.2 | +1.1 | +1.0 |
| 188 | YPR156C | *TPO3* | -1.3 | -2.8 | -1.8 | -1.6 | +2.2 | +1.9 | +1.1 | -1.1 |
| 189 | YPR183W | *DPM1* | -1.7 | -2.5 | -1.3 | -1.3 | +1.4 | +1.3 | -1.1 | +1.3 |
| 190 | YBL005W-B | *YBL005W-B* | +1.3 | +1.7 | +2.6 | +3.2 | +1.5 | +1.1 | -1.2 | +1.2 |
| 191 | YDR056C | *YDR056C* | +1.5 | +1.8 | +2.5 | +2.6 | +1.8 | +1.3 | -1.1 | -1.0 |
| 192 | YDR210C-D | *YDR210C-D* | +1.6 | +1.7 | +2.3 | +3.1 | +1.5 | +1.1 | -1.1 | +1.5 |
| 193 | YDR541C | *YDR541C* | -1.0 | +1.7 | +3.3 | +3.0 | +1.4 | -1.0 | -1.1 | -1.2 |
| 194 | YGR135W | *PRE9* | +2.4 | +2.2 | +2.0 | +1.8 | +2.2 | +1.3 | -1.1 | +1.1 |
| 195 | YHR214C-B | *YHR214C-B* | +1.6 | +1.7 | +2.3 | +2.5 | +1.5 | +1.2 | -1.1 | +1.4 |
| 196 | YJR072C | *NPA3* | -1.1 | +1.8 | +2.3 | +1.6 | +1.2 | -1.1 | -1.4 | -1.4 |
| 197 | YLR157C-B | *YLR157C-B* | +1.1 | +1.6 | +2.6 | +2.3 | +1.5 | -1.0 | -1.2 | +1.0 |
| 198 | YLR199C | *PBA1* | +1.9 | +2.3 | +2.3 | +1.6 | +1.7 | +1.2 | +1.0 | -1.1 |
| 199 | YLR227W-B | *YLR227W-B* | +1.5 | +1.5 | +2.3 | +2.7 | +1.5 | -1.0 | -1.4 | +1.5 |
| 200 | YML039W | *YML039W* | +1.6 | +1.9 | +2.6 | +2.6 | +1.6 | +1.1 | -1.2 | +1.2 |
| 201 | YMR102C | *YMR102C* | +1.8 | +1.6 | +2.2 | +2.9 | +1.6 | +1.4 | -1.3 | -1.2 |
| 202 | YMR271C | *URA10* | +1.8 | +1.4 | +1.9 | +3.1 | +4.7 | +3.4 | +3.6 | +6.9 |
| 203 | YMR314W | *PRE5* | +2.2 | +2.1 | +1.9 | +1.5 | +1.7 | +1.2 | -1.2 | -1.1 |
| 204 | YPR158C-D | *YPR158C-D* | +1.7 | +1.7 | +2.5 | +2.8 | +1.6 | +1.2 | -1.1 | +1.6 |
| 205 | YBR128C | *ATG14* | +2.1 | +1.5 | +1.5 | +1.4 | +3.3 | +2.3 | +1.6 | +8.9 |
| 206 | YCL055W | *KAR4* | +1.9 | +2.1 | +2.2 | +1.8 | +2.8 | +1.4 | -1.0 | -1.1 |
| 207 | YDL059C | *RAD59* | +1.7 | +2.1 | +1.8 | +2.8 | +1.1 | +1.3 | -1.2 | +1.6 |
| 208 | YOL023W | *IFM1* | +2.1 | +2.0 | +1.7 | +2.8 | +2.2 | +1.7 | +1.0 | +1.2 |
| 209 | YBR010W | *HHT1* | -1.6 | -2.0 | -1.6 | -1.1 | +1.4 | +1.2 | -1.2 | +1.3 |
| 210 | YBR068C | *BAP2* | -1.2 | -1.5 | -1.1 | -1.3 | +2.6 | +1.6 | +1.4 | +1.0 |
| 211 | YBR116C | *YBR116C* | -1.4 | -1.6 | -1.1 | -1.5 | +2.2 | +1.8 | +1.4 | +2.0 |
| 212 | YBR145W | *ADH5* | +1.1 | -1.4 | -1.7 | -1.4 | +3.2 | +2.1 | +1.7 | +1.8 |
| 213 | YDL170W | *UGA3* | -1.2 | -1.3 | -1.3 | -1.5 | +2.2 | +1.6 | +1.1 | +1.4 |
| 214 | YDL182W | *LYS20* | -2.6 | -1.6 | -1.4 | -1.3 | +2.2 | +1.8 | +2.9 | +1.9 |
| 215 | YDR034C | *LYS14* | -1.2 | -1.5 | -1.2 | -1.2 | +2.1 | +1.4 | +1.1 | +1.3 |
| 216 | YDR046C | *BAP3* | -1.0 | -1.6 | -1.5 | -1.5 | +2.6 | +1.9 | +1.2 | -1.1 |
| 217 | YDR127W | *ARO1* | -1.5 | -1.7 | -1.2 | -1.5 | +2.1 | +1.6 | +1.2 | +1.3 |
| 218 | YDR234W | *LYS4* | -2.6 | -1.6 | -1.0 | -1.0 | +.8 | +1.3 | +1.1 | +1.3 |
| 219 | YDR492W | *IZH1* | -2.3 | -1.8 | -1.0 | -1.3 | +1.8 | +1.3 | +1.5 | +1.2 |
| 220 | YEL071W | *DLD3* | -1.6 | -1.1 | -1.4 | -2.0 | +2.0 | +1.3 | +1.1 | +1.1 |
| 221 | YER090W | *TRP2* | -1.5 | -1.5 | -1.2 | -1.2 | +2.1 | +1.5 | +1.3 | +1.3 |
| 222 | YER174C | *GRX4* | -1.8 | -1.6 | -1.3 | -1.5 | +2.0 | +1.4 | +1.1 | -1.0 |
| 223 | YGL028C | *SCW11* | -1.1 | -1.9 | -1.5 | -1.2 | +2.2 | +1.5 | +1.1 | -1.4 |
| 224 | YHR020W | *YHR020W* | -2.2 | -1.8 | -1.2 | -1.4 | +1.7 | +1.3 | +1.1 | +1.2 |
| 225 | YHR122W | *YHR122W* | -3.4 | -1.3 | -1.1 | -1.7 | +1.6 | +1.2 | +1.2 | +1.6 |
| 226 | YHR162W | *YHR162W* | -1.1 | -1.4 | -1.3 | -1.3 | +2.5 | +1.7 | +1.3 | +1.6 |
| 227 | YHR183W | *GND1* | -1.2 | -2.2 | -1.5 | -1.3 | +1.8 | +1.4 | +1.2 | +1.5 |
| 228 | YHR204W | *MNL1* | -2.1 | -2.2 | -1.2 | -1.3 | +1.3 | +1.3 | +1.1 | +1.2 |
| 229 | YJR160C | *MPH3* | +1.2 | -1.5 | -1.9 | -1.3 | +2.3 | +1.7 | +1.7 | +1.4 |
| 230 | YKL143W | *LTV1* | -2.9 | -1.4 | -1.2 | -1.3 | +1.5 | +1.3 | -1.0 | +1.3 |
| 231 | YKL182W | *FAS1* | -1.3 | -1.8 | -1.0 | -1.1 | +2.2 | +1.6 | +1.5 | +1.4 |
| 232 | YLR249W | *YEF3* | -2.2 | -1.7 | -1.1 | -1.7 | +1.8 | +1.2 | +1.1 | -1.2 |
| 233 | YLR355C | *ILV5* | -2.3 | -2.0 | -1.3 | -1.3 | +1.5 | +1.1 | +1.1 | +1.4 |
| 234 | YLR441C | *RPS1A* | -1.5 | -2.2 | -1.2 | -1.4 | +2.0 | +1.5 | +1.1 | +1.3 |
| 235 | YLR449W | *FPR4* | -2.6 | -1.4 | -1.2 | -1.4 | +1.5 | +1.1 | -1.3 | +1.0 |
| 236 | YML116W | *ATR1* | -1.2 | -1.2 | -1.0 | -1.7 | +2.1 | +1.7 | +1.4 | +1.1 |
| 237 | YMR106C | *YKU80* | -1.3 | -1.5 | -1.4 | -1.2 | +2.6 | +1.7 | +1.0 | +1.1 |
| 238 | YMR142C | *RPL13B* | -1.8 | -2.1 | -1.4 | -1.1 | +1.6 | +1.3 | +1.0 | +1.4 |
| 239 | YMR229C | *RRP5* | -3.1 | -1.4 | -1.0 | -1.3 | +1.8 | +1.3 | +1.0 | +1.2 |
| 240 | YMR230W | *RPS10B* | -1.6 | -2.1 | -1.5 | -1.1 | +1.4 | +1.2 | +1.2 | +1.4 |
| 241 | YNL014W | *HEF3* | -2.0 | -1.5 | -1.2 | -1.2 | +1.7 | +1.1 | -1.1 | +1.9 |
| 242 | YNL302C | *RPS19B* | -2.0 | -1.8 | -1.4 | -1.3 | +1.7 | +1.2 | +1.0 | +1.4 |
| 243 | YNL303W | *YNL303W* | -1.2 | -1.5 | +1.1 | -1.2 | +1.9 | +1.6 | +1.0 | +2.3 |
| 244 | YOL140W | *ARG8* | -2.0 | -1.9 | -1.1 | -1.1 | +2.2 | +1.8 | +2.2 | +3.0 |
| 245 | YOR006C | *TSR3* | -2.4 | -1.4 | -1.2 | -1.2 | +1.4 | +1.0 | -1.1 | +1.3 |
| 246 | YOR056C | *NOB1* | -2.2 | -1.2 | -1.0 | -1.3 | +1.4 | +1.2 | +1.0 | +1.2 |
| 247 | YOR347C | *PYK2* | -1.6 | -2.0 | -1.1 | -1.3 | +1.8 | +1.2 | -1.1 | +1.2 |
| 248 | YPL177C | *CUP9* | -1.3 | -1.6 | -1.3 | -1.2 | +2.0 | +1.8 | +1.0 | +2.1 |
| 249 | YPL212C | *PUS1* | -2.9 | -1.4 | -1.0 | -1.5 | +1.4 | +1.2 | +1.1 | +1.1 |
| 250 | YPL264C | *YPL264C* | +1.0 | -1.7 | -1.1 | -1.4 | +2.5 | +1.5 | +1.6 | +1.2 |
| 251 | YPR058W | *YMC1* | -1.5 | -1.4 | +1.0 | -1.5 | +2.4 | +1.6 | +1.4 | +1.3 |
| 252 | YPR130C | *YPR130C* | -1.4 | -2.3 | -1.2 | -1.3 | +1.6 | +1.2 | +1.1 | +1.3 |
| 253 | YPR132W | *RPS23B* | -1.4 | -2.0 | -1.3 | -1.3 | +1.7 | +1.4 | +1.2 | +1.4 |
| 254 | YBL027W | *RPL19B* | -2.0 | -2.4 | -1.8 | -1.4 | +1.2 | +1.1 | -1.4 | +1.1 |
| 255 | YBL092W | *RPL32* | -2.4 | -2.9 | -1.9 | -1.6 | +1.1 | -1.0 | -1.5 | -1.1 |
| 256 | YBR028C | *YBR028C* | -2.1 | -1.9 | -1.5 | -1.8 | +1.6 | +1.2 | -1.0 | -1.0 |
| 257 | YBR031W | *RPL4A* | -2.7 | -3.2 | -1.6 | -1.9 | +1.2 | +1.0 | -1.2 | -1.0 |
| 258 | YBR048W | *RPS11B* | -2.6 | -3.6 | -1.9 | -1.4 | +1.1 | +1.0 | -1.3 | +1.0 |
| 259 | YBR191W | *RPL21A* | -1.9 | -2.4 | -1.5 | -1.6 | +1.4 | +1.2 | -1.2 | +1.1 |
| 260 | YBR191W-A | *YBR191W-A* | -2.0 | -2.6 | -1.7 | -1.7 | +1.4 | +1.3 | -1.0 | +1.2 |
| 261 | YBR249C | *ARO4* | -2.1 | -3.5 | -2.3 | -2.6 | +1.7 | +1.3 | +1.1 | +1.3 |
| 262 | YCL030C | *HIS4* | -2.9 | -4.5 | -3.6 | -3.1 | +2.6 | +1.9 | +1.4 | +1.7 |
| 263 | YCR005C | *CIT2* | -2.1 | -1.2 | -2.6 | -3.2 | +2.2 | +1.2 | +1.2 | +1.2 |
| 264 | YDL051W | *LHP1* | -3.0 | -1.3 | -1.7 | -1.9 | +1.2 | +1.0 | -1.4 | +1.2 |
| 265 | YDL075W | *RPL31A* | -2.4 | -2.6 | -2.0 | -1.5 | +1.1 | -1.0 | -1.4 | +1.2 |
| 266 | YDL213C | *NOP6* | -5.0 | -1.6 | -1.3 | -1.5 | +1.1 | -1.2 | -1.5 | -1.3 |
| 267 | YDR025W | *RPS11A* | -2.4 | -3.3 | -1.8 | -1.6 | +1.1 | +1.1 | -1.2 | +1.1 |
| 268 | YDR035W | *ARO3* | -1.9 | -2.6 | -2.2 | -2.7 | +1.9 | +1.5 | +1.1 | +1.4 |
| 269 | YDR037W | *KRS1* | -2.5 | -1.8 | -1.3 | -1.8 | +1.5 | +1.1 | -1.1 | -1.0 |
| 270 | YDR133C | *YDR133C* | -2.0 | -3.6 | -4.2 | -3.2 | +1.3 | +1.2 | -1.1 | -1.0 |
| 271 | YDR222W | *YDR222W* | -3.2 | -2.8 | -2.0 | -1.7 | +1.5 | +1.4 | -1.1 | -1.1 |
| 272 | YDR447C | *RPS17B* | -1.9 | -2.2 | -1.7 | -1.6 | +1.5 | +1.3 | -1.2 | +1.3 |
| 273 | YDR471W | *RPL27B* | -2.3 | -2.1 | -1.7 | -1.5 | +1.3 | +1.2 | -1.3 | +1.2 |
| 274 | YER052C | *HOM3* | -3.3 | -2.4 | -1.7 | -1.7 | +2.0 | +1.5 | +1.3 | +1.1 |
| 275 | YER055C | *HIS1* | -3.4 | -2.5 | -2.1 | -2.3 | +1.9 | +1.3 | +1.2 | +1.3 |
| 276 | YER056C-A | *RPL34A* | -2.4 | -2.5 | -1.6 | -1.4 | +1.1 | -1.0 | -1.4 | +1.2 |
| 277 | YER069W | *ARG5,6* | -3.2 | -4.1 | -2.5 | -2.2 | +2.3 | +1.7 | +2.3 | +4.3 |
| 278 | YER073W | *ALD5* | -3.6 | -1.8 | -1.2 | -1.2 | +1.9 | +1.3 | +1.1 | +1.5 |
| 279 | YER102W | *RPS8B* | -1.9 | -2.4 | -1.7 | -1.7 | +1.4 | +1.2 | -1.2 | -1.0 |
| 280 | YER110C | *KAP123* | -2.7 | -1.9 | -1.1 | -1.6 | +1.6 | +1.3 | +1.1 | -1.1 |
| 281 | YER126C | *NSA2* | -4.2 | -1.6 | -1.4 | -1.8 | +1.0 | -1.0 | -1.1 | -1.2 |
| 282 | YER131W | *RPS26B* | -2.3 | -2.7 | -1.8 | -1.4 | +1.3 | +1.2 | -1.3 | +1.4 |
| 283 | YER145C | *FTR1* | -3.3 | -2.2 | -1.7 | -3.7 | +1.0 | +1.0 | -1.1 | -1.1 |
| 284 | YER175C | *TMT1* | -2.7 | -2.9 | -2.2 | -2.4 | +2.5 | +2.0 | +1.4 | +1.8 |
| 285 | YFL034C-A | *RPL22B* | -3.1 | -2.5 | -1.8 | -1.2 | +1.5 | +1.3 | -1.2 | +1.5 |
| 286 | YGL055W | *OLE1* | -3.5 | -2.5 | -1.1 | -1.3 | +1.7 | +1.2 | +1.1 | +1.2 |
| 287 | YGL103W | *RPL28* | -1.6 | -2.8 | -1.8 | -1.5 | +1.4 | +1.2 | +1.3 | +1.1 |
| 288 | YGL117W | *YGL117W* | -5.7 | -5.7 | -5.7 | -4.9 | +2.2 | +1.6 | -1.0 | +1.3 |
| 289 | YGL147C | *RPL9A* | -1.7 | -2.8 | -2.0 | -1.5 | +1.7 | +.2 | -1.1 | +1.1 |
| 290 | YGL189C | *RPS26A* | -2.4 | -2.7 | -2.0 | -1.4 | +1.2 | +1.1 | -1.4 | +1.3 |
| 291 | YGR034W | *RPL26B* | -2.0 | -2.2 | -1.6 | -1.6 | +1.3 | +1.2 | -1.3 | +1.1 |
| 292 | YGR085C | *RPL11B* | -2.7 | -2.5 | -1.7 | -1.7 | +1.4 | +1.1 | -1.2 | +1.1 |
| 293 | YGR148C | *RPL24B* | -2.0 | -2.6 | -1.8 | -1.4 | +1.4 | +1.2 | -1.3 | +1.2 |
| 294 | YGR214W | *RPS0A* | -2.2 | -2.8 | -1.5 | -1.6 | +1.4 | +1.2 | -1.0 | +1.0 |
| 295 | YGR245C | *SDA1* | -5.6 | -1.3 | -1.5 | -1.7 | +1.2 | -1.2 | -1.4 | -1.0 |
| 296 | YHL015W | *RPS20* | -2.4 | -3.0 | -2.0 | -2.1 | +1.2 | -1.1 | -1.5 | -1.0 |
| 297 | YHL033C | *RPL8A* | -2.8 | -4.0 | -2.1 | -2.1 | +1.2 | +1.0 | -1.2 | +1.1 |
| 298 | YHR010W | *RPL27A* | -2.2 | -2.2 | -1.5 | -1.4 | +1.4 | +1.2 | -1.1 | +1.3 |
| 299 | YIL018W | *RPL2B* | -2.2 | -3.2 | -1.5 | -1.7 | +1.5 | +1.2 | -1.0 | +1.0 |
| 300 | YIL052C | *RPL34B* | -2.5 | -2.6 | -1.8 | -1.5 | +1.1 | -1.0 | -1.5 | +1.4 |
| 301 | YIL068C | *SEC6* | -2.2 | -2.3 | -1.6 | -1.6 | +1.4 | +1.2 | -1.2 | +1.2 |
| 302 | YIL069C | *RPS24B* | -2.3 | -2.5 | -1.6 | -1.5 | +1.4 | +1.3 | -1.1 | +1.2 |
| 303 | YIL131C | *FKH1* | -2.6 | -1.9 | -1.5 | -1.2 | +1.3 | -1.1 | -1.1 | +1.2 |
| 304 | YIL133C | *RPL16A* | -2.6 | -2.8 | -1.4 | -1.7 | +1.6 | +1.2 | +1.2 | +1.1 |
| 305 | YIL148W | *RPL40A* | -1.8 | -2.3 | -1.7 | -1.6 | +1.3 | +1.1 | -1.3 | +1.3 |
| 306 | YJL088W | *ARG3* | -9.1 | -5.8 | -4.9 | -2.6 | +2.1 | +1.5 | +2.4 | +3.5 |
| 307 | YJL177W | *RPL17B* | -2.2 | +2.6 | -1.6 | -1.5 | +1.4 | +1.2 | +1.1 | +1.1 |
| 308 | YJL200C | *ACO2* | -3.2 | -2.3 | -1.6 | -1.5 | +1.8 | +1.1 | -1.0 | +1.3 |
| 309 | YJR070C | *LIA1* | -4.4 | -1.7 | -1.4 | -1.4 | -1.0 | -1.1 | -1.3 | -1.2 |
| 310 | YJR109C | *CPA2* | -4.7 | -4.2 | -4.3 | -4.2 | +2.3 | +1.6 | +1.3 | +1.6 |
| 311 | YJR123W | *RPS5* | -2.1 | -3.2 | -1.9 | -1.9 | +1.6 | +1.2 | -1.3 | -1.0 |
| 312 | YJR145C | *RPS4A* | -2.0 | -2.7 | -1.6 | -1.8 | +1.5 | +1.2 | -1.1 | -1.0 |
| 313 | YKL029C | *MAE1* | -4.9 | -2.9 | -2.0 | -2.9 | -1.1 | +1.1 | -1.6 | -1.0 |
| 314 | YKL069W | *YKL069W* | -2.2 | -2.2 | -1.7 | -1.1 | +1.4 | -1.0 | -1.2 | -1.0 |
| 315 | YKL180W | *RPL17A* | -2.2 | -2.8 | -1.6 | -1.4 | +1.4 | +1.3 | -1.0 | +1.3 |
| 316 | YKR094C | *RPL40B* | -2.0 | -2.1 | -1.8 | -1.5 | +1.3 | +1.2 | -1.4 | +1.2 |
| 317 | YLL045C | *RPL8B* | -2.7 | -3.8 | -2.1 | -2.1 | +1.3 | +1.1 | -1.2 | -1.0 |
| 318 | YLR029C | *RPL15A* | -2.2 | -2.9 | -1.6 | -1.7 | +1.6 | +1.3 | -1.2 | +1.3 |
| 319 | YLR040C | *YLR040C* | -2.4 | -2.4 | -1.8 | -2.0 | +1.5 | +1.2 | -1.3 | -1.1 |
| 320 | YLR048W | *RPS0B* | -2.3 | -3.3 | -1.5 | -1.7 | +1.5 | +1.3 | +1.0 | +1.2 |
| 321 | YLR106C | *MDN1* | -2.3 | -1.5 | -1.4 | -2.4 | +1.8 | +1.2 | -1.3 | -1.6 |
| 322 | YLR130C | *ZRT2* | -1.9 | -3.9 | -3.6 | -3.6 | +1.9 | +1.6 | +1.3 | +1.1 |
| 323 | YLR150W | *STM1* | -1.6 | -2.4 | -1.8 | -1.5 | +1.3 | +1.1 | -1.4 | +1.2 |
| 324 | YLR175W | *CBF5* | -3.7 | -1.4 | -1.3 | -1.5 | +1.4 | -1.0 | -1.1 | +1.2 |
| 325 | YLR197W | *NOP56* | -3.8 | -1.4 | -1.2 | -1.6 | +1.4 | +1.0 | -1.0 | +1.2 |
| 326 | YLR340W | *RPP0* | -2.1 | -3.6 | -1.7 | -1.9 | +1.3 | +1.1 | -1.1 | -1.1 |
| 327 | YLR341W | *SPO77* | -1.7 | -2.5 | -1.6 | -1.9 | +1.3 | +1.2 | -1.0 | -1.2 |
| 328 | YLR367W | *RPS22B* | -2.3 | -2.1 | -1.5 | -1.7 | +1.5 | +1.3 | -1.1 | +1.0 |
| 329 | YLR406C | *RPL31B* | -2.1 | -2.5 | -1.7 | -1.5 | +1.3 | +1.1 | -1.3 | +1.5 |
| 330 | YMR058W | *FET3* | -4.3 | -4.5 | -2.5 | -5.1 | +1.3 | +1.6 | +1.1 | +1.1 |
| 331 | YMR062C | *ARG7* | -2.0 | -2.5 | -2.2 | -2.5 | +2.2 | +1.5 | +1.1 | +1.3 |
| 332 | YMR116C | *ASC1* | -2.0 | -3.1 | -1.9 | -1.7 | +1.4 | +1.1 | +1.1 | -1.0 |
| 333 | YMR121C | *RPL15B* | -2.3 | -2.8 | -2.0 | -2.1 | +1.4 | +1.1 | -1.3 | -1.0 |
| 334 | YMR194W | *RPL36A* | -2.6 | -2.5 | -1.7 | -1.3 | +1.0 | -1.1 | -1.4 | +1.3 |
| 335 | YMR195W | *ICY1* | -3.9 | -2.2 | -3.1 | -3.1 | +2.0 | +1.3 | +1.0 | +1.4 |
| 336 | YMR242C | *RPL20A* | -2.0 | -2.5 | -1.6 | -1.6 | +1.4 | +1.1 | -1.2 | +1.1 |
| 337 | YMR243C | *ZRC1* | -2.5 | -1.8 | -1.1 | -1.5 | +1.6 | +1.2 | -1.0 | -1.0 |
| 338 | YMR321C | *YMR321C* | -2.6 | -2.2 | -2.0 | -3.1 | +1.6 | -1.0 | -1.0 | +1.1 |
| 339 | YNL049C | *SFB2* | -2.7 | -2.2 | -1.0 | -1.3 | +2.4 | -1.1 | -1.2 | +1.0 |
| 340 | YNL096C | *RPS7B* | -3.6 | -2.9 | -1.7 | -1.8 | +1.1 | +1.1 | -1.5 | +1.1 |
| 341 | YNL112W | *DBP2* | -5.5 | -2.5 | -3.5 | -3.1 | +1.4 | -1.1 | -1.0 | -1.3 |
| 342 | YNR057C | *BIO4* | -2.2 | -3.2 | -2.7 | -4.1 | +1.2 | +1.1 | -1.3 | -1.4 |
| 343 | YOL040C | *RPS15* | -2.5 | -3.6 | -2.0 | -1.8 | +1.1 | +1.0 | -1.3 | -1.2 |
| 344 | YOL058W | *ARG1* | -5.8 | -10.5 | -7.1 | -6.3 | +1.8 | +1.4 | +1.5 | +2.3 |
| 345 | YOL121C | *RPS19A* | -2.2 | -2.2 | -1.8 | -1.9 | +1.3 | +1.0 | -1.4 | +1.0 |
| 346 | YOL127W | *RPL25* | -2.3 | -2.4 | -1.9 | -1.8 | +1.2 | +1.0 | -1.4 | +1.2 |
| 347 | YOR130C | *ORT1* | -2.2 | -2.0 | -1.8 | -1.9 | +1.8 | +1.2 | +1.1 | +1.1 |
| 348 | YOR202W | *HIS3* | -2.5 | -3.0 | -2.1 | -2.2 | +1.7 | +1.3 | +1.1 | +1.4 |
| 349 | YOR222W | *ODC2* | -2.1 | -4.0 | -3.3 | -3.2 | +1.8 | +1.2 | +1.0 | +1.1 |
| 350 | YOR234C | *RPL33B* | -2.2 | -2.3 | -1.7 | -1.8 | +1.2 | +1.0 | -1.2 | +1.0 |
| 351 | YOR302W | *YOR302W* | -4.1 | -2.1 | -1.6 | -1.2 | +1.2 | +1.0 | -1.0 | +1.9 |
| 352 | YOR303W | *CPA1* | -3.6 | -2.0 | -1.6 | -1.3 | +1.4 | +1.3 | -1.0 | +1.8 |
| 353 | YOR312C | *RPL20B* | -2.1 | -2.4 | -1.7 | -1.8 | +1.3 | +1.1 | -1.3 | +1.1 |
| 354 | YOR355W | *GDS1* | -3.5 | -3.8 | -1.7 | -1.8 | +1.4 | +1.1 | -1.0 | +1.3 |
| 355 | YPL131W | *RPL5* | -2.3 | -2.9 | -1.4 | -1.3 | +1.4 | +1.2 | -1.1 | +1.1 |
| 356 | YPL143W | *RPL33A* | -2.3 | -2.4 | -1.6 | -1.5 | +1.2 | +1.0 | -1.2 | +1.1 |
| 357 | YPL198W | *RPL7B* | -2.1 | -3.2 | -2.0 | -1.9 | +1.3 | +1.1 | -1.2 | -1.1 |
| 358 | YPL249C-A | *RPL36B* | -2.5 | -2.4 | -1.8 | -1.4 | -1.0 | -1.1 | -1.5 | +1.3 |
| 359 | YPL250C | *ICY2* | -5.9 | -2.7 | -3.7 | -3.4 | +1.2 | +1.3 | -1.2 | +1.4 |
| 360 | YPL252C | *YAH1* | -3.5 | -2.8 | -2.0 | -1.7 | -1.1 | -1.1 | -1.2 | +1.4 |
| 361 | YPL273W | *SAM4* | -3.1 | -2.6 | -2.6 | -3.5 | +1.7 | +1.2 | -1.1 | +1.1 |
| 362 | YPL274W | *SAM3* | -2.1 | -2.8 | -3.0 | -5.1 | +2.2 | +1.8 | +1.6 | +1.3 |
| 363 | YPR102C | *RPL11A* | -2.4 | -2.4 | -1.5 | -1.6 | +1.6 | +1.3 | -1.1 | +1.3 |
| 364 | YPR157W | *TDA6* | -2.1 | -3.7 | -2.5 | -1.8 | +2.3 | +2.1 | -1.1 | -1.5 |
| 365 | YNL311C | *YNL311C* | -2.0 | -1.7 | -1.3 | -1.9 | +1.4 | +1.1 | -1.1 | +1.0 |
