# Supplementary material for: Comparative transcriptome profiling analyses during the lag phase uncover YAP1, PDR1, PDR3, RPN4, and HSF1 as key regulatory genes in genomic adaptation to the lignocellulose derived inhibitor HMF for Saccharomyces cerevisiae
Source: BMC Genomics. 2010 Nov 24;11:660. doi: 10.1186/1471-2164-11-660 (PMC3091778; doi:10.1186/1471-2164-11-660)
Supplement: Additional file 2 — Gene Ontology (GO) categories and terms for significantly repressed genes by HMF (30 mM) challenge in Saccharomyces cerevisiae NRRL Y-12632. [file 1471-2164-11-660-S2.DOC]

Additional file 2. Gene Ontology (GO) categories and terms for significantly repressed genes by HMF challenge during the lag phase in *Saccharomyces cerevisiae*

| GO ID | GO term | Gene(s) |
| --- | --- | --- |
| Cellular component | | |
| GO:0005737 | Cytoplasm | *RPL19B, RPS8A, RPL23A, RPL32, YBR028C, RPL4A, RPS11B, RPL19A, TKL2, ADH5, RTC2, RPS6B, RPS9B, RPL21A, PYC2, ARO4, ILV6, CIT2, PSA1, RPL31A, RPL13A, RPS16B, DLD1, LYS20, GGC1, FMP45, RPS11A, ARO3, KRS1, BAP3, RPS13, ARO1, HOM2, YDR222W, LYS4, TRP4, RPP2B, EFT2, RPL12B, RPS17B, RPS18A, RPL27B, IZH1, RPL12A, DLD3, HOM3, RPL34A, ARG5, 6, ALD5, RPS24A, SER3, TRP2, RPS8B, KAP123, RPL23B, RPS26B, TMT1, RPL22B, RPL2A, RPL30, RPL24A, OLE1, RPL7A, RPL28, RPS2, MET13, RPL1B, RPL9A, ARO2, RPS26A, ARO8, RPL26B, RPL11B, TPO2, RPL24B, RPS0A, BIO2, RPS20, RPL8A, RPL27A, ARG4, YHR020W, YHR122W, YHR162W, GND1, RPS4B, MNL1, RPL2B, RPL34B, SEC6, RPS24B, RPL16A, RPL40A, ARG3, RPL17B, ACO2, ILV3, BNA1, LIA1, CPA2, RPS5, STR2, RPS4A, MAE1, YKL069W, LTV1, RPL17A, FAS1, RPL40B, RPL8B, RPL15A, RPS0B, MDN1, STM1, RPS31, YEF3, RPP0, RPL26A, ILV5, RPS22B, RPL31B, RPS1A, RPL6B, RPS17A, RPS18B, RPS1B, ATR1, ARG7, ASC1, RPL15B, RPL13B, RPS16A, RPL36A, ICY1, RPS10B, RPL20A, ZRC1, YMR315W, HEF3, SFB2, RPL9B, RPL16B, RPS7B, LEU4, DBP2, SSB2, RPL18B, RPS19B, YNL311C, BIO4, RPS15, ARG1, RPL18A, RPS19A, RPL25, ARG8, TSR3, NOB1, RPL3, RPS7A, ORT1, EFT1, ODC2, RPL33B, YOR302W, CPA1, RPL20B, PYK2, GDS1, RPL21B, RPS9A, RPS6A, RPL5, RPL33A, RPL7B, RPL1A, RPL36B, YAH1, SAM4, YMC1, RPL11A, RPS23B, TPO3, DPM1* |
| GO:0005840 | Ribosome | *RPL19B, RPS8A, RPL23A, RPL32, RPL4A, RPS11B, RPL19A, RPS6B, RPS9B, RPL21A, RPL31A, RPL13A, RPS16B, RPS11A, RPS13, RPP2B, EFT2, RPL12B, RPS17B, RPS18A, RPL27B, RPL12A, RPL34A, RPS24A, RPS8B, RPL23B, RPS26B, RPL22B, RPL2A, RPL30, RPL24A, RPL7A, RPL28, RPS2, RPL1B, RPL9A, RPS26A, RPL26B, RPL11B, RPL24B, RPS0A, RPS20, RPL8A, RPL27A, YHR020W, RPS4B, RPL2B, RPL34B, RPS24B, RPL16A, RPL40A, RPL17B, RPS5, RPS4A, RPL17A, RPL40B, RPL8B, RPL15A, RPS0B, STM1, RPS31, YEF3, RPP0, RPL26A, RPS22B, RPL31B, RPS1A, RPL6B, RPS17A, RPS18B, RPS1B, ASC1, RPL15B, RPL13B, RPS16A, RPL36A, RPS10B, RPL20A, HEF3, RPL9B, RPL16B, RPS7B, RPL18B, RPS19B, YNL311C, RPS15, RPL18A, RPS19A, RPL25, RPL3, RPS7A, EFT1, RPL33B, RPL20B, RPL21B, RPS9A, RPS6A, RPL5, RPL33A, RPL7B, RPL1A, RPL36B, RPL11A, RPS23B* |
| GO:0005634 | Nucleus | *HHT1, TKL2, ADH5, ARO4, LHP1, UGA3, LYS20, NOP6, LYS14, ARO3, HOM2, TRP4, KAP123, NSA2, GRX4, RPL28, SDA1, PCL5, YHR122W, FKH1, ZAP1, LIA1, STR2, YKL069W, LTV1, MDN1, CBF5, NOP56, FPR4, YKU80, RRP5, YMR315W, HHT2, DBP2, TSR3, NOB1, GDS1, CUP9, RPL7B, PUS1, SAM4* |
| GO:0005739 | Mitochondrion | *RTC2, ILV6, CIT2, DLD1, LYS20, GGC1, FMP45, ARO3, BAP3, LYS4, RPS18A, ARG5, 6, ALD5, RPS24A, MET13, BIO2, YHR162W, GND1, ACO2, ILV3, MAE1, FAS1, RPL40B, MDN1, ILV5, RPS18B, ARG7, LEU4, DBP2, ARG8, ORT1, ODC2, PYK2, GDS1, YAH1, YMC1, DPM1* |
| GO:0016020 | Membrane | *AST1, BAP2, DLD1, GGC1, FMP45, BAP3, IZH1, KAP123, FTR1, OLE1, TPO2, DUR3, MPH3, LTV1, TRK2, ZRT2, ATR1, HXT2, FET3, ICY1, SFB2, PNS1, ODC2, YPL264C, SAM3, TPO3, DPM1* |
| GO:0005575 | Cellular component unknown | *YBR116C, YBR191W-A, YBR300C, YDR133C, PES4, YGL117W, YGL149W, YGL230C, YGR291C, YIL059C, NIT1, YIL165C, YJL220W, YLR112W, YLR152C, YMR321C, YNL303W, HIS3, ICY2, YPR130C, TDA6* |
| GO:0005886 | Plasma membrane | *BAP2, BAP3, FTR1, TPO2, DUR3, MPH3, TRK2, ZRT2, ATR1, HXT2, FET3, PNS1, SAM3, TPO3* |
| GO:0005624 | Membrane fraction | *MAL32, FMP45, SSB1, HOM2, MAL12, HXT2, SSB2, PNS1, TPO3* |
| GO:0005730 | Nucleolus | *LHP1, NOP6, CBF5, NOP56, RRP5, RPL7B* |
| GO:0005773 | Vacuole | *TPO2, ATR1, ICY1, ZRC1, TPO3* |
| GO:0005740 | Mitochondrial envelope | *DLD1, GGC1, ORT1, ODC2, DPM1* |
| GO:0005783 | Endoplasmic reticulum | *IZH1, OLE1, MNL1, DPM1* |
| GO:0012505 | Endomembrane system | *KAP123, OLE1, SFB2, DPM1* |
| GO:0005694 | Chromosome | *HHT1, FPR4, YKU80, HHT2* |
| GO:0005938 | Cell cortex | *FMP45, SEC6* |
| GO:0005618 | Cell wall | *SCW11, YLR040C* |
| GO:0005777 | Peroxisome | *CIT2* |
| GO:0005794 | Golgi apparatus | *SFB2* |
| GO:0016023 | Cytoplasmic membrane-bounded vesicle | *SFB2* |
| GO:0030427 | Site of polarized growth | *SEC6* |
| GO:0005933 | Cellular bud | *SEC6* |
| Other | Other | *HIS4, HIS1, HIS5, SPO77* |
| Not yet annotated | Not yet annotated | *YER186W-A* |
| Biological process | | |
| GO:0006412 | Translation | *RPL19B, RPS8A, RPL23A, RPL32, RPL4A, RPS11B, RPL19A, RPS6B, RPS9B, RPL21A, RPL31A, RPL13A, RPS16B, SSB1, RPS11A, KRS1, RPS13, RPP2B, EFT2, RPL12B, RPS17B, RPS18A, RPL27B, RPL12A, RPL34A, RPS24A, RPS8B, RPL23B, RPS26B, RPL22B, RPL2A, RPL30, RPL24A, RPL7A, RPL28, RPS2, RPL1B, RPL9A, RPS26A, RPL26B, RPL11B, RPL24B, RPS0A, RPS20, RPL8A, RPL27A, YHR020W, RPS4B, RPL2B, RPL34B, RPS24B, RPL16A, RPL40A, RPL17B, RPS5, RPS4A, RPL17A, RPL40B, RPL8B, RPL15A, RPS0B, STM1, RPS31, YEF3, RPP0, RPL26A, RPS22B, RPL31B, RPS1A, RPL6B, RPS17A, RPS18B, RPS1B, ASC1, RPL15B, RPL13B, RPS16A, RPL36A, RPS10B, RPL20A, HEF3, RPL9B, RPL16B, RPS7B, SSB2, RPL18B, RPS19B, RPS15, RPL18A, RPS19A, RPL25, RPL3, RPS7A, EFT1, RPL33B, YOR302W, RPL20B, RPL21B, RPS9A, RPS6A, RPL5, RPL33A, RPL7B, RPL1A, RPL36B, RPL11A, RPS23B* |
| GO:0042254 | Ribosome biogenesis | *RPS8A, RPS11B, RPS6B, RPS9B, RPS16B, NOP6, RPS11A, RPS13, RPL12B, RPS17B, RPS18A, RPL12A, RPS24A, RPS8B, NSA2, RPS26B, RPL30, RPS2, RPS26A, RPL11B, RPS0A, SDA1, RPS20, RPS24B, RPL40A, RPS5, LTV1, RPL40B, RPS0B, MDN1, RPS31, CBF5, NOP56, RPP0, RPS1A, RPL6B, RPS17A, RPS18B, RPS1B, RPS16A, RRP5, RPS10B, RPS7B, DBP2, RPS19B, RPS15, RPS19A, RPL25, NOB1, RPL3, RPS7A, RPS9A, RPS6A, RPL5, RPL11A, RPS23B* |
| GO:0006519 | Cellular amino acid and derivative metabolic process | *ADH5, ARO4, ILV6, HIS4, CIT2, UGA3, LYS20, LYS14, ARO3, KRS1, ARO1, HOM2, LYS4, TRP4, HOM3, HIS1, ARG5, 6, SER3, TRP2, MET13, ARO2, ARO8, ARG4, YHR020W, HIS5, ARG3, ILV3, LIA1, CPA2, STR2, MAE1, ILV5, ARG7, ZRC1, LEU4, ARG1, ARG8, ORT1, HIS3, CPA1, SAM4* |
| GO:0016070 | RNA metabolic process | *RPS8A, RPS11B, RPS6B, RPS9B, LHP1, RPS16B, UGA3, RPS11A, KRS1, RPS13, RPS18A, RPS24A, RPS8B, NSA2, RPL30, RPS0A, RPS20, YHR020W, RPS24B, FKH1, ZAP1, RPS0B, MDN1, RPS31, CBF5, NOP56, RPS1A, FPR4, RPS18B, RPS1B, YKU80, RPS16A, RRP5, DBP2, NOB1, RPS9A, RPS6A, CUP9, PUS1, RPS23B* |
| GO:0006810 | Transport | *AST1, BAP2, GGC1, BAP3, RPS18A, KAP123, RPS26B, FTR1, RPS2, RPS26A, TPO2, RPS0A, SDA1, DUR3, SEC6, RPS5, MPH3, LTV1, RPS0B, ZRT2, RPS18B, ATR1, HXT2, FET3, RPS10B, ZRC1, SFB2, RPS19B, RPS15, RPS19A, ORT1, ODC2, SAM3, YMC1, TPO3* |
| GO:0008150 | Biological process unknown | *YBR028C, YBR116C, RTC2, YBR191W-A, YBR300C, YDR133C, YDR222W, TMT1, PES4, YGL117W, YGL149W, YGL230C, YGR291C, YHR162W, YIL059C, NIT1, YIL165C, ACO2, YJL220W, YLR040C, YLR112W, YLR152C, ICY1, YMR321C, YNL303W, TSR3, PNS1, ICY2, YPL264C, YPR130C, TDA6* |
| GO:0042221 | Response to chemical stimulus | *HOM2, IZH1, HOM3, RPL23B, GRX4, GND1, RPL2B, YKL069W, LTV1, ATR1, FET3, ASC1* |
| GO:0044262 | Cellular carbohydrate metabolic process | *TKL2, ADH5, PYC2, MAL32, CIT2, PSA1, DLD1, MAL12, GND1, DPM1* |
| GO:0051186 | Cofactor metabolic process | *TKL2, ADH5, PYC2, BIO2, GND1, BNA1, ZRC1, YMR315W, BIO4, YAH1* |
| GO:0019725 | Cellular homeostasis | *GGC1, IZH1, FTR1, GRX4, ZAP1, TRK2, FET3, ZRC1, CUP9* |
| GO:0046483 | Heterocycle metabolic process | *HIS4, TRP4, HIS1, TRP2, BIO2, HIS5, BIO4, HIS3, YAH1* |
| GO:0051276 | Chromosome organization | *HHT1, YHR020W, YHR122W, FKH1, STM1, FPR4, YKU80, HHT2* |
| GO:0006350 | Transcription | *UGA3, ARG5, 6, PCL5, FKH1, ZAP1, FPR4, YKU80, CUP9* |
| GO:0006766 | Vitamin metabolic process | *TKL2, ADH5, PYC2, BIO2, GND1, BNA1, YMR315W, BIO4* |
| GO:0006725 | Cellular aromatic compound metabolic process | *ARO4, ARO3, ARO1, TRP4, TRP2, ARO2, ARO8, SAM4* |
| GO:0007049 | Cell cycle | *HHT1, SDA1, YHR122W, SEC6, FKH1, HHT2* |
| GO:0006464 | Protein modification process | *PSA1, RPL40A, LIA1, RPL40B, FPR4, DPM1* |
| GO:0006950 | Response to stress | *GRX4, GND1, YKL069W, LTV1, STM1, YKU80* |
| GO:0044255 | Cellular lipid metabolic process | *IZH1, OLE1, FAS1, DPM1* |
| GO:0006091 | Generation of precursor metabolites and energy | *ADH5, DLD1, GDS1* |
| GO:0007005 | Mitochondrion organization | *GGC1, OLE1, ILV5* |
| GO:0070271 | Protein complex biogenesis | *SFB2, NOB1, CUP9* |
| GO:0006259 | DNA metabolic process | *FKH1, STM1, YKU80* |
| GO:0007010 | Cytoskeleton organization | *SDA1, LIA1* |
| GO:0000910 | Cytokinesis | *SCW11, SEC6* |
| GO:0045333 | Cellular respiration | *DLD1, GDS1* |
| GO:0006457 | Protein folding | *SSB1, SSB2* |
| GO:0016050 | Vesicle organization | *SEC6, SFB2* |
| GO:0030435 | Sporulation resulting in formation of a cellular spore | *FMP45, SPO77* |
| GO:0016044 | Membrane organization | *SEC6, SFB2* |
| GO:0032196 | Transposition | *HHT1, ASC1* |
| GO:0007165 | Signal transduction | *STM1, ASC1* |
| GO:0031505 | Fungal-type cell wall organization | *FMP45, ARG7* |
| GO:0044257 | Cellular protein catabolic process | *MNL1, YNL311C* |
| GO:0016192 | Vesicle-mediated transport | *SEC6, SFB2* |
| GO:0007059 | Chromosome segregation | *YHR122W* |
| GO:0032989 | Cellular component morphogenesis | *SEC6* |
| GO:0007124 | Pseudohyphal growth | *FKH1* |
| GO:0007114 | Cell budding | *SEC6* |
| Other | Other | *DLD3, ALD5, PYK2* |
| Not yet annotated | Not yet annotated | *YER186W-A* |
| Molecular function | | |
| GO:0005198 | Structural molecule activity | *RPL19B, RPS8A, RPL23A, RPL32, RPL4A, RPS11B, RPL19A, RPS6B, RPS9B, RPL21A, RPL31A, RPL13A, RPS16B, RPS11A, RPS13, RPP2B, RPL12B, RPS17B, RPS18A, RPL27B, RPL12A, RPL34A, RPS24A, RPS8B, RPL23B, RPS26B, RPL22B, RPL2A, RPL30, RPL24A, RPL7A, RPL28, RPS2, RPL1B, RPL9A, RPS26A, RPL26B, RPL11B, RPL24B, RPS0A, RPS20, RPL8A, RPL27A, RPS4B, RPL2B, RPL34B, RPS24B, RPL16A, RPL40A, RPL17B, RPS5, RPS4A, RPL17A, RPL40B, RPL8B, RPL15A, RPS0B, RPS31, RPP0, RPL26A, RPS22B, RPL31B, RPS1A, RPL6B, RPS17A, RPS18B, RPS1B, RPL15B, RPL13B, RPS16A, RPL36A, RPS10B, RPL20A, RPL9B, RPL16B, RPS7B, RPL18B, RPS19B, RPS15, RPL18A, RPS19A, RPL25, RPL3, RPS7A, RPL33B, RPL20B, RPL21B, RPS9A, RPS6A, RPL5, RPL33A, RPL7B, RPL1A, RPL36B, RPL11A, RPS23B* |
| GO:0003674 | Molecular function unknown | *AST1, YBR116C, RTC2, YBR191W-A, YBR300C, FMP45, YDR133C, YDR222W, NSA2, PES4, YGL117W, YGL149W, YGL230C, SDA1, YGR291C, YHR122W, YHR162W, YIL059C, YIL165C, YJL220W, LTV1, YLR040C, YLR112W, YLR152C, NOP56, SPO77, ICY1, YMR321C, YNL303W, TSR3, PNS1, GDS1, ICY2, YPL264C, YPR130C, TDA6* |
| GO:0016740 | Transferase activity | *YBR028C, TKL2, ARO4, ILV6, CIT2, PSA1, LYS20, ARO3, ARO1, TRP4, HOM3, HIS1, ARG5, 6, TMT1, ARO8, BIO2, HIS5, ARG3, STR2, FAS1, ARG7, LEU4, ARG8, PYK2, SAM4, DPM1* |
| GO:0003723 | RNA binding | *LHP1, NOP6, RPS13, RPL30, RPL24A, RPL28, RPS2, RPL26B, RPL24B, RPL16A, RPL15A, RPP0, RPL26A, RPL6B, YKU80, RPL15B, RPL36A, RRP5, RPL16B, RPL25, NOB1, RPS9A, RPL5, RPL36B* |
| GO:0016491 | Oxidoreductase activity | *ADH5, HIS4, DLD1, ARO1, HOM2, DLD3, ARG5, 6, ALD5, SER3, GRX4, OLE1, MET13, ARO2, GND1, BNA1, LIA1, MAE1, YKL069W, FAS1, ILV5, FET3, YMR315W, YAH1* |
| GO:0005215 | Transporter activity | *BAP2, GGC1, BAP3, KAP123, FTR1, TPO2, DUR3, MPH3, TRK2, ZRT2, ATR1, HXT2, FET3, ZRC1, ORT1, ODC2, SAM3, YMC1, TPO3* |
| GO:0016787 | Hydrolase activity | *MAL32, HIS4, SSB1, SCW11, MAL12, MNL1, NIT1, MDN1, YEF3, HEF3, DBP2, SSB2, NOB1* |
| GO:0016829 | Lyase activity | *ARO1, LYS4, TRP2, ARO2, ARG4, ACO2, ILV3, FAS1, CBF5, HIS3* |
| GO:0003677 | DNA binding | *HHT1, UGA3, LYS14, FKH1, ZAP1, STM1, YKU80, HHT2, CUP9* |
| GO:0016874 | Ligase activity | *PYC2, KRS1, YHR020W, CPA2, BIO4, ARG1, CPA1* |
| GO:0005515 | Protein binding | *SSB1, SEC6, FPR4, ASC1, SSB2, YNL311C* |
| GO:0030528 | Transcription regulator activity | *UGA3, LYS14, FKH1, ZAP1, CUP9* |
| GO:0016853 | Isomerase activity | *CBF5, FPR4, PUS1* |
| GO:0030234 | Enzyme regulator activity | *ILV6, PCL5, ASC1* |
| GO:0004672 | Protein kinase activity | *YBR028C* |
| GO:0016779 | Nucleotidyltransferase activity | *PSA1* |
| GO:0004871 | Signal transducer activity | *ASC1* |
| GO:0004386 | Helicase activity | *DBP2* |
| GO:0045182 | Translation regulator activity | *YOR302W* |
| Other | Other | *EFT2, IZH1, SFB2, EFT1* |
| Not yet annotated | Not yet annotated | *YER186W-A* |
