# Supplementary figures and images for: Comparative transcriptome profiling analyses during the lag phase uncover YAP1, PDR1, PDR3, RPN4, and HSF1 as key regulatory genes in genomic adaptation to the lignocellulose derived inhibitor HMF for Saccharomyces cerevisiae
Source: BMC Genomics. 2010 Nov 24;11:660. doi: 10.1186/1471-2164-11-660 (PMC3091778; doi:10.1186/1471-2164-11-660)

## Slide 1
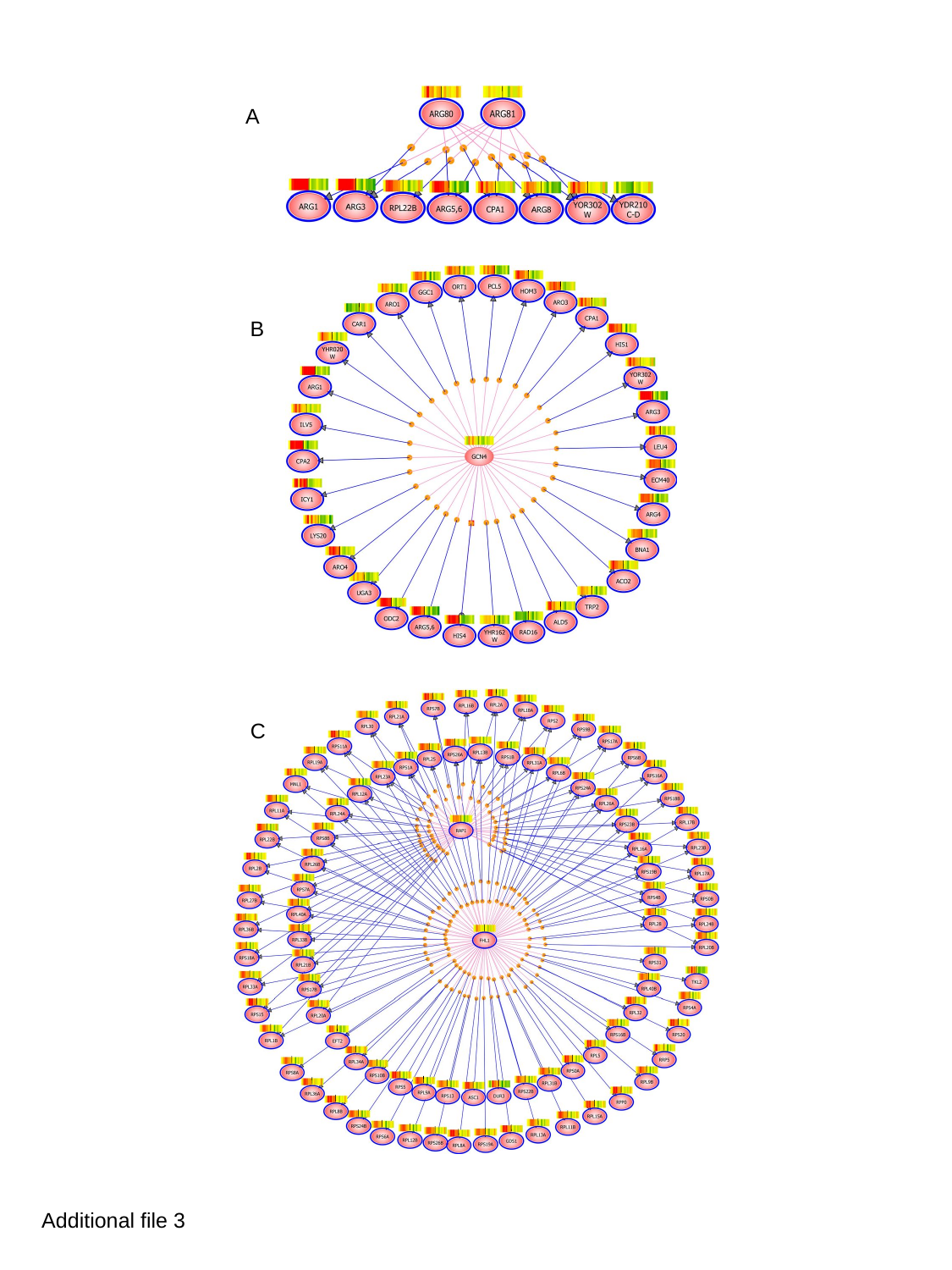

A
B
C
Additional file 3

Supplement: Additional file 3 — Regulatory interaction networks for repressed genes under HMF stress. [file 1471-2164-11-660-S3.PPT]
