# Supplementary material for: Comparative transcriptome profiling analyses during the lag phase uncover YAP1, PDR1, PDR3, RPN4, and HSF1 as key regulatory genes in genomic adaptation to the lignocellulose derived inhibitor HMF for Saccharomyces cerevisiae
Source: BMC Genomics. 2010 Nov 24;11:660. doi: 10.1186/1471-2164-11-660 (PMC3091778; doi:10.1186/1471-2164-11-660)
Supplement: Additional file 4 — Protein binding motifs and binding elements for significantly induced genes by HMF challenge during the lag phase in Saccharomyces cerevisiae. [file 1471-2164-11-660-S4.DOC]

Additional file 4. Protein binding motifs and binding elements for significantly induced genes by HMF challenge during the lag phase in *Saccharomyces cerevisiae*

| ORF | Gene | Yap1p response elements | Pleiotropic drug response elements | Proteasome-associated control elements | Heat shock elements |
| --- | --- | --- | --- | --- | --- |
| YCR105W | *ADH7* | TTACTAA, TGACTAA | - | - | NGAANNTTCN |
| YGL157W | *ARI1* | - | - | - | NTTCNNGAAN |
| YOL151W | *GRE2* | TKACAAA | TCCGTGGA, TCCACGGA | - | NGAANNTTCN |
| YPL171C | *OYE3* | TTACTAA, TKACAAA | - | - | NTTCNNGAAN |
| YOR374W | *ALD4* | TTACTAA, TKACAAA | - | - | - |
| YML130C | *ERO1* | TTACTAA, TGACTCA | - | - | NGAANNNNNNNGAANNNNNNNGAAN |
| YLR142W | *PUT1* | - | - | - | - |
| PUT2 | *YHR037W* | TTACTAA, TKACAAA | - | - | - |
| YOR153W | *PDR5* | TTACTAA | TCCGCGGA, TCCGTGGA, TCCACGGA | - | NGAANNTTCN |
| YPL058C | *PDR12* | TTACTAA | - | - | NGAANNTTCN |
| YDR406W | *PDR15* | - | TCCGCGGA | - | - |
| YGR281W | *YOR1* | TGACTAA | TCCGTGGA, TCCACGGA | - | - |
| YDR011W | *SNQ2* | TTACTAA,  TGACTAA | TCCGCGGA,  TCCGTGGA,  TCCACGGA,  TCCGCGCA | - | - |
| YOR049C | *RSB1* | TTACTAA | TCCGCGGA | - | - |
| YLR099C | *ICT1* | - | TCCGTGGA, TCCACGGA | - | NTTCNNGAAN |
| YLL028W | *TPO1* | TGACTCA, TGACTAA | TCCGTGGA,  TCCACGGA | - | - |
| YOR273C | *TPO4* | - | TCCGTGGA, TCCACGGA | - | NTTCNNGAAN |
| YER142C | *MAG1* | TTACTAA, TKACAAA | - | - | - |
| YER143W | *DDI1* | TKACAAA | - | - | - |
| YGL062W | *PYC1* | - | - | - | - |
| YML125C | *PGA3* | - | TCCGCGGA | - | - |
| YGR035C | *YGR035C* | - | TCCGTGGA, TCCACGGA | - | NGAANNTTCN |
| YLL056C | *YLL056C* | TTACTAA, TKACAAA | TCCGTGGA, TCCACGGA | - | NTTCNNGAAN |
| YER012W | *PRE1* | - | - | GGTGGCAAA | NTTCNNGAAN |
| YJL001W | *PRE3* | TGACTCA, TKACAAA | - | GGTGGCAAA | NGAANNTTCN |
| YOL038W | *PRE6* | - | - | GGTGGCAAA | NGAANNTTCN |
| YBL041W | *PRE7* | - | - | GGTGGCAAA | - |
| YOR362C | *PRE10* | - | - | GGTGGCAAA | - |
| YDL007W | *RPT2* | TTACTAA | - | GGTGGCAAA | - |
| YDR394W | *RPT3* | TKACAAA | - | GGTGGCAAA | NTTCNNGAAN |
| YOR259C | *RPT4* | TKACAAA | - | GGTGGCAAA | - |
| PDR427W | *RPN9* | TGACTCA, TGACTAA, TKACAAA |  | GGTGGCAAA | NTTCNNGAAN |
| YFR052W | *RPN12* | - | - | GGTGGCAAA | NTTCNNGAAN |
| YER094C | *PUP3* | - | - | GGTGGCAAA | NTTCNNGAAN |
| YFL044C | *OTU1* | - | - | - | NGAANNTTCN |
| YHL030W | *ECM29* | - | - | GGTGGCAAA | - |
| YOR007C | *SGT2* | TKACAAA | - | GGTGGCAAA | NGAANNTTCN |
| YBL058W | *SHP1* | TTACTAA, TKACAAA | - | - | NGAANNTTCN, NTTCNNGAAN |
| YOR052C | *YOR052C* | - | - | GGTGGCAAA | - |
| YNL155W | *YNL155W* | TTACTAA, TGACTAA, TKACAAA | - | GGTGGCAAA | NTTCNNGAAN |
| YBR072W | *HSP26* | - | - | - | NGAANNTTCN, NTTCNNGAAN |
| YER103W | *SSA4* | TKACAAA | - | - | NTTCNNGAAN, NGAANNNNNNNGAANNNNNNNGAAN |
| YLR089C | *ALT1* | TKACAAA | TCCGCGGG | - | - |
| YPL156C | *PRM4* | - | - | - | - |
| YDL021W | *GPM2* | TTACTAA, TKACAAA | - | - | NGAANNTTCN, NTTCNNGAAN |
| YJR010W | *MET3* | TKACAAA | - | - | - |
| YKL001C | *MET14* | TKACAAA | - | - | NTTCNNGAAN |
| YCL064C | *CHA1* | - | TCCGCGCA | - | NTTCNNGAAN |
| YPL111W | *CAR1* | TGACTCA | - | - | - |
| YBL078C | *ATG8* | - | - | - | - |
| YBR114W | *RAD16* | TGACTCA | - | - | - |
| YOR009W | *TIR4* | - | TCCGCGCA | - | NTTCNNGAAN |
| YAR073W | *IMD1* | TGACTAA, TKACAAA | - | - | NGAANNTTCN, NTTCNNGAAN |
| YBR170C | *NPL4* | TGACTAA, TKACAAA | - | - | - |
| YDR515W | *SLF1* | TTACTAA | - | - | NTTCNNGAAN |
| YOR306C | *MCH5* | TKACAAA | - | - | NGAANNTTCN, NTTCNNGAAN |
| YBL101W-A | *YBL101W-A* | - | - | - | - |
| YBR062C | *YBR062C* | - | - | - | - |
| YBR255C-A | *YBR255C-A* | - | - | - | NGAANNTTCN |
| YDR210W-B | *YDR210W-B* | TKACAAA | - | - | NTTCNNGAAN |
| YDR316W-B | *YDR316W-B* | - | - | - | NGAANNTTCN, NTTCNNGAAN, NGAANNTTCNNGAAN |
| YDR365W-B | *YDR365W-B* | - | - | - | NGAANNTTCN |
| YGR111W | *YGR111W* | - | - | - | NTTCNNGAAN |
| YHR138C | *YHR138C* | TGACTAA | - | - | NTTCNNGAAN |
| YKR011C | *YKR011C* | TKACAAA | - | - | NTTCNNGAAN |
| YOR059C | *YOR059C* | TKACAAA | - | - | - |
| YBL107C | *YBL107C* | - | - | - | NGAANNTTCN |
| YDR034W-B | *YDR034W-B* | - | - | - | NGAANNTTCN, NTTCNNGAAN |
| YER137C | *YER137C* | TKACAAA | - | - | - |
| YML007W | *YAP1* | TKACAAA | - | GGTGGCAAA | NTTCNNGAAN |
| YDL020C | *RPN4* | TTACTAA, TKACAAA | TCCGTGGA, TCCACGGA |  | NGAANNTTCN, NTTCNNGAAN, NGAANNTTCNNGAAN, NGAANNNNNNNGAANNNNNNNGAAN |
| Total | | 41 | 16 | 16 | 42 |
