# Supplementary material for: Comparative transcriptome profiling analyses during the lag phase uncover YAP1, PDR1, PDR3, RPN4, and HSF1 as key regulatory genes in genomic adaptation to the lignocellulose derived inhibitor HMF for Saccharomyces cerevisiae
Source: BMC Genomics. 2010 Nov 24;11:660. doi: 10.1186/1471-2164-11-660 (PMC3091778; doi:10.1186/1471-2164-11-660)
Supplement: Additional file 5 — Primers used for mRNA expression analysis for Saccharomyces cerevisiae by real-time qRT-PCR using SYBR Green I. [file 1471-2164-11-660-S5.DOC]

Additional File 5. Primers used for mRNA expression analysis for *Saccharomyces cerevisiae* by real-time qRT-PCR using SYBR Green I

| ID | Sequence 5’-3’ | Direction | Amplicon (bp) | Gene / ORF (Note) |
| --- | --- | --- | --- | --- |
| B2M_L | agcgtcctccaaagattcaa | Forward |  |  |
| B2M_R | tccccattcttcagcaaatc | Reverse | 127 | *B2M* (Control) |
| MSG_L | gatgagcacagccttgtgaa | Forward |  |  |
| MSG_R | cctccacgttcttggtgagt | Reverse | 112 | *MSG* (Control) |
| CAB_L | agacagcactcccatggttc | Forward |  |  |
| CAB_ R | aatcccatcagtgccatctc | Reverse | 109 | *CAB* (Control) |
| RBS1_L | gcttggaattcgagttggag | Forward |  |  |
| RBS1_R | gagaagcatcagtgcaacca | Reverse | 123 | *RBS1* (Control) |
| ACTB_L | gctctcttccagccttcctt | Forward |  |  |
| ACTB_R | tagaggtccttgcggatgtc | Reverse | 104 | *ACTB* (Control) |
| PDR1a_L | aatccggatctcccaagtct | Forward |  |  |
| PDR1a_R | ttgtttctcactggggaagg | Reverse | 110 | *PDR1*/YGL013C |
| PDR3_L | atactgccgaacggagaaga | Forward |  |  |
| PDR3_R | ctgaaatccttcggcaagag | Reverse | 130 | *PDR3*/YBL005W |
| PDR5_L | tgacgcttttgcatcagttc | Forward |  |  |
| PDR5_R | gagaaaaccgcgacaatgtt | Reverse | 150 | *PDR5*/YOR153W |
| PDR10_L | ctcgggtttgaatcaaggaa | Forward |  |  |
| PDR10_R | aaaagggaagattggctcgt | Reverse | 135 | *PDR10*/YOR328W |
| PDR15_L | ggttggtccatttggatttg | Forward |  |  |
| PDR15_R | ttgggcacaagggaatctac | Reverse | 99 | *PDR15*/YDR406W |
| YOR1b_L | ttccctgcaattttggctat | Forward |  |  |
| YOR1b_R | atgaaaaacccaccgaaaaa | Reverse | 98 | *YOR1*/YGR281W |
| SNQ2_L | tatcaaaagctggccaatcc | Forward |  |  |
| SNQ2_R | gtttgtccacccttcctcaa | Reverse | 103 | *SNQ2*/YDR011W |
| ICT1_L | actactgcagacgccaaggt | Forward |  |  |
| ICT1_R | tgtggaaatgccactggtta | Reverse | 139 | *ICT1*/YLR099C |
| DDI1_L | atacaggggctcaaacaacg | Forward |  |  |
| DDI1_R | attttgacttgggcttggtg | Reverse | 148 | *DDI1*/YER143W |
| TPO1_L | tcggtatgatggtgtgtgct | Forward |  |  |
| TPO1_R | ggatagtagcccgtccaaca | Reverse | 169 | *TPO1*/YLL028W |
| GRE2_L | cgtatcggaggccagattta | Forward |  |  |
| GRE2_R | atgggtagcaccagaacctg | Reverse | 112 | *GRE2*/YOL151W |
| PGA3a_L | tgtggacctgatggaatgaa | Forward |  |  |
| PGA2a_R | acttgatcgtcgccagaact | Reverse | 95 | PGA3/YML125C |
| YMR102C_L | acaccaagggaaagcatgtc | Forward |  |  |
| YMR102C_R | agcaccagaaaaagcctcaa | Reverse | 129 | YMR102C |
| YGR035C_L | attcattcatgcgtgccata | Forward |  |  |
| YGR035C_R | cattttcagcagcagctttg | Reverse | 150 | YGR035C |
